# Analysis of Fbox substrate adapter proteins using *ProteoSync*, a program for projection of evolutionary conservation onto protein atomic coordinates

**DOI:** 10.1016/j.csbj.2025.09.012

**Published:** 2025-09-11

**Authors:** Elliot Sicheri, Daniel Mao, Michael Tyers, Frank Sicheri

**Affiliations:** a Lunenfeld-Tanenbaum Research Institute, Sinai Health System, Toronto, Ontario M5G 1×5, Canada; b Department of Molecular Genetics, University of Toronto, Toronto, Ontario M5S 1A8, Canada; c SickKids Hospital, Toronto, Ontario M5G 1×8, Canada; d Department of Biochemistry, University of Toronto, Toronto, Ontario M5S 1A8, Canada

**Keywords:** Evolutionary conservation, Sequence alignment tool, Protein structural analysis

## Abstract

The projection of conservation onto the surface of a protein’s 3D structure is a powerful way of inferring functionally important regions. For this reason, we created ProteoSync, a Python program that semi-automates the process. The program creates an annotated sequence alignment of orthologs from a diverse set of selectable species and enables the fast projection of amino acid conservation onto a predicted or known 3D model in PyMOL ^1^. As a test case, we used ProteoSync to analyze a subset of 31 F-box proteins, which function as substrate recognition subunits for a large family of Cul1-based E3 ubiquitin ligases. We correctly identified known substrate interaction surfaces for 11 F-box members with previously solved structures. We also identified likely ligand binding sites for 16 other members, thus demonstrating ProteoSync’s utility for discovering conserved, functionally relevant surfaces.

## Graphical abstract

**Figure:**
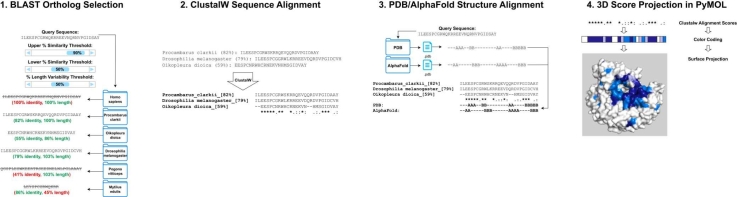

## 1 Introduction

At present, the workflow for identifying conserved and functionally important surfaces on a protein’s structure can be involved and tedious. First, one must manually search through candidate species for likely orthologs of the protein of interest and then use them to generate a multi protein sequence alignment to determine conservation. Then one manually projects this conservation onto a protein’s structure, followed by visual inspection of the results. Since the process is iterative and requires inspection of the alignments to filter out redundant sequences and non-orthologs (for example structural homologs), the tediousness of the process is amplified. There are many tools that can perform individual parts of this pipeline. For example, the National Center for Biotechnology Information (NCBI) provides the online tool BLAST [2] to find similar protein sequences to an input sequence and COBALT [3] for the automated creation of alignments from input sequences, while the program ProtSkin can take in a sequence alignment and projects it onto a 3D model [4]. The program ConSurf [5], on the other hand, automates all steps in the process. It uses a selectable search algorithm to locate orthologs, generates a sequence alignment, uses statistical modelling to calculate conservation scores and presents 3-dimensional projections onto a protein surface. However, the ConSurf web server can take significant time to analyze a single sequence, which hinders the processing of many proteins in bulk, and lacks the ability to limit the analysis to specific taxonomical groups of interest. For this reason, we created ProteoSync, a Python program that creates annotated sequence alignments of likely orthologs from a diverse, customizable set of species, and that enables the convenient projection of amino acid conservation scores onto a 3D model using the structure visualization program PyMOL [1]. ProteoSync is available for download from GitHub (https://github.com/ElliotSicheri/ProteoSync) alongside a starter set of 135 diverse species sequence databases. The package is composed of a directory which contains the python scripts, setup and usage instructions, database files, and subdirectories for outputs. A detailed description of the downloaded contents is provided in the instructions folder. The program uses several dependencies and the installation instructions for these are detailed in Supplementary Information 1.

## 2 Methods

ProteoSync accepts a sequence or UniProt ID [6], [7] for the query protein, upper and lower % identity threshold to filter likely functional orthologs and reduce sequence redundancy, a % length threshold to help filter fragmented sequences, a list of species to analyze entered through a checkbox menu, and lastly a run name. If a UniProt ID is entered, the program will download the protein’s sequence from UniProt and use that as the query sequence. The program operates in 4 steps: BLAST ortholog search, CLUSTALW sequence alignment, PDB and AlphaFold structure analysis, and PyMOL script generation for surface projection.

**Table 1:** List of species available in the included starter species database set.

**Taxonomic Group**	**Species**	**Taxonomic Group**	**Species**	**Taxonomic Group**	**Species**
Porifera	*Sycon ciliatum*	Reptilia (reptiles and birds)	*Pogona vitticeps*	Bacteria	*Bacillus subtilis*
	*Amphimedon queenslandica*		*Crotalus tigris*		*Mycobacterium tuberculosis*
Hemichordata	*Ptychodera flava*		*Trachemys scripta elegans*		*Mycoplasmoides genitalium*
	*Saccoglossus kowalevskii*		*Anolis carolinensis*		*Staphylococcus aureus*
Mollusks	*Mercenaria mercenaria*		*Gallus gallus*		*Streptomyces coelicolor*
	*Mytilus edulis*		*Columba livia*		*Synechocystis*
	*Octopus bimaculoides*		*Taeniopygia guttata*		*Agrobacterium radiobacter*
	*Octopus sinensis*	Mammalia	*Equus caballus*		*Aliivibrio fischeri*
Annelids	*Dimorphilus gyrociliatus*		*Otolemur garnetti*		*Azotobacter vinelandii*
	*Lamellibrachia satsuma*		*Bos taurus*		*Caulobacter vibrioides*
Cnidaria	*Stylophora pistillata*		*Myotis myotis*		*Cereibacter sphaeroides*
	*Nematostella vectensis*		*Rattus norvegicus*		*Escherichia coli*
Cephalochordata	*Branchiostoma floridae*		*Neosciurus carolinensis*		*Myxococcus xanthus*
	*Branchiostoma lanceolatum*		*Homo sapiens*	*Viridiplantae*	*Arabidopsis thaliana*
Tunicata	*Ciona intestinalis*		*Macaca mulatta*		*Brassica rapa*
	*Oikopleura dioica*		*Ursus arctos*		*Chara braunii*
Amphibia	*Xenopus laevis*		*Orcinus orca*		*Chlamydomonas reinhardtii*
	*Xenopus tropicalis*		*Monodelphis domestica*		*Helianthus annuus*
	*Geotrypetes seraphini*		*Canis lupus*		*Nymphaea colorata*
Protista	*Naegleria gruberi*		*Mus musculus*		*Oryza sativa*
	*Dictyostelium discoideum*		*Sus scrofa*		*Physcomitrium patens*
	*Entamoeba histolytica*		*Felis catus*		*Solanum lycopersicum*
	*Guillardia theta*	Platyhelminthes	*Schistosoma japonicum*		*Triticum aestivum*
	*Leishmania major*		*Echinococcus granulosis*		*Vigna angularis*
	*Trypanosoma brucei*	Insecta	*Drosophilia melanogaster*		*Zea mays*
	*Emiliania huxleyi*		*Danaus plexippus*		*Amborella trichopoda*
	*Giardia duodenalis*		*Solenopsis invicta*		*Beta vulgaris*
	*Spironucleus salmonicida*	Arachnida	*Parasteatoda tepidariorum*		*Glycine max*
	*Capsaspora owczarzaki*		*Ixodes scapularis*		*Lactuca sativa*
	*Albugo laibachii*	Crustacea	*Penaeus japonicus*		*Musa acuminata*
	*Bigelowiella natans*		*Penaeus vannamei*		*Nicotiana attenuata*
	*Phytophthora lateralis*		*Homarus americanus*		*Ostreococcus lucimarinus*
	*Plasmodium falciparum*		*Portunus trituberculatus*		*Prunus persica*
	*Tetrahymena thermophila*		*Procambarus clarkii*		*Selaginella moellendorffii*
	*Reticulomyxa filosa*	Fish	*Eptatretus burgeri*	Echinodermata	*Acanthaster planci*
Archaea	*Archaeoglobus fulgidus*		*Latimeria chalumnae*		*Patiria miniata*
	*Halobacterium salinarum*		*Callorhinchus milli*		*Lytechinus variegatus*
	*Methanosarcina mazei*		*Scyliorhynus torazame*	Fungi	*Neurospora crassa*
	*Picrophilus oshimae*		*Petromyzon marinus*		*Saccharomyces cerevisiae*
	*Pyrococcus furiosus*		*Anguilla anguilla*		*Schizosaccharomyces pombe*
	*Thermoplasma volcanium*		*Takifugu rubripes*		*Coprinopsis cinerea*
	*Nanoarchaeum equitans*		*Danio rerio*	Nemotoda	*Caenorhabditis elegans*
	*Caldivirga maquilingensis*		*Cynoglossus semilaevis*		*Meloidogyne enterolobii*
	*Pyrobaculum aerophilum*		*Salmo salar*		
	*Saccharolobus solfataricus*		*Oncorhynchus nerka*		
	*Sulfurisphaera tokodaii*		*Protopterus annectens*		

### 2.1 Searching for protein orthologs via BLAST

**Fig. 1:**
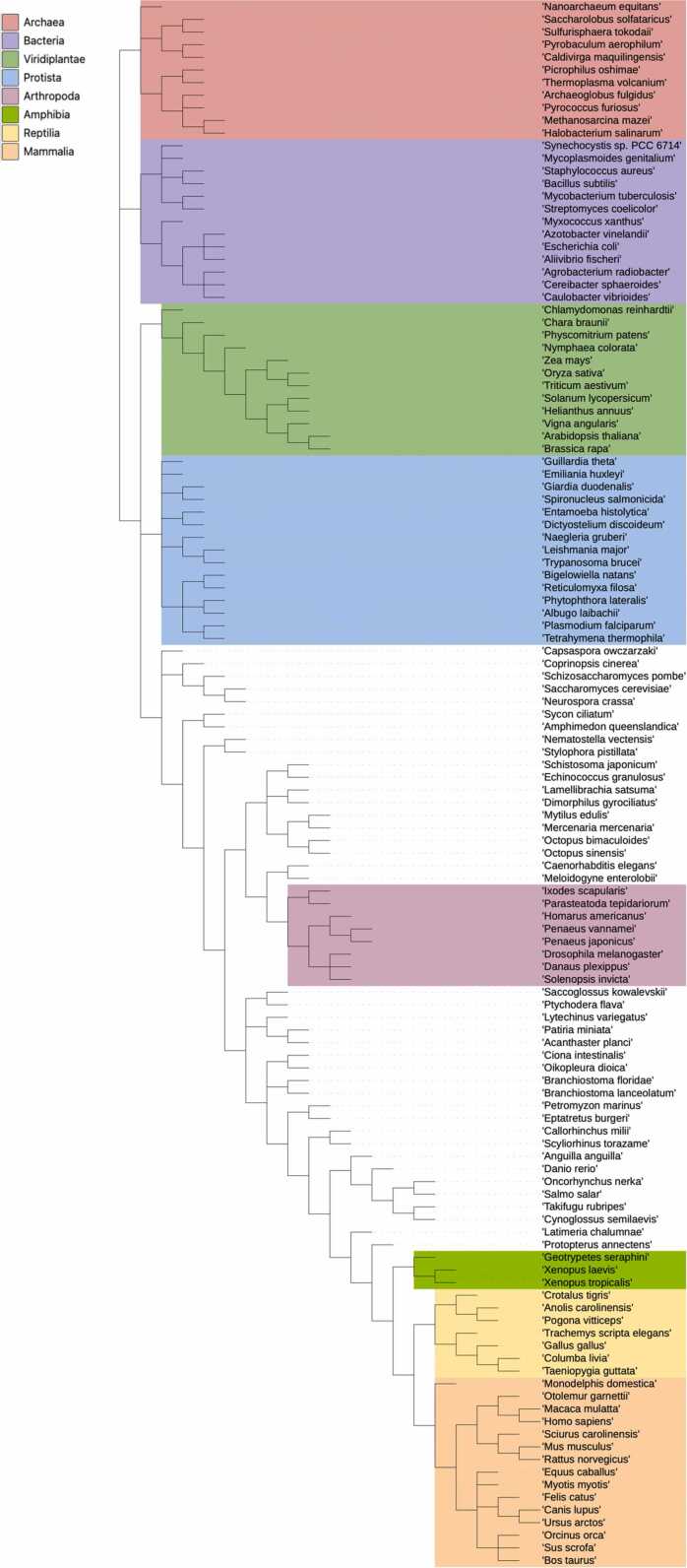
Taxonomy tree of the 135 species included in the ProteoSync starter dataset.

ProteoSync starts by using the BLAST search algorithm [2] to search a series of databases, each containing every protein sequence encoded in a particular species’ genome. Each species database provided in the software release was obtained either from NCBI [8] or Ensembl [9] as described in Supplementary Information 2. We provide a starter dataset of 135 species that covers a wide diversity of organisms including mammals, plants, bacteria and archaea (Fig. 1**)**. The user can also expand the set of species beyond the base set by following instructions described in Supplementary Information 2. The arrangement of the dataset subdirectories is automatically reflected in the species checkbox menu, allowing the user to organize the datasets by phylogeny.

The BLAST search returns a single sequence most similar to the query sequence from each species database, which is most likely but not necessarily the direct functional ortholog to the query protein. Taking only the most similar sequence from each species helps to minimize the problem of including structural (i.e. non-functional) orthologs from the same species, and prevents the inclusion of redundant duplicate sequences that may be present in the datasets. However, it has the disadvantage that it excludes duplicated gene products with the same function. For example, in human there are two isoforms of the E3 ubiquitin ligase substrate adapter βTrCP, namely βTrCP 1 and βTrCP 2 (aka FBWX1 and FBWX11), which are 78 % identical and encoded as separate genes. While both use a common highly conserved surface to bind phospho-motifs in their substrates [10], the two proteins would not be treated as orthologs by ProteoSync.

As there is a possibility that the sequence identified is a protein homolog (same protein fold but divergent function) rather than a protein ortholog (same protein fold and same function), the user can define a lower identity threshold ranging from 0 % to 100 % to help filter out non-desirable protein sequences. All identified sequences with a % identity to the query sequence above the threshold are selected for sequence alignment. Note that the identity threshold must be optimized for each query protein by iteratively running ProteoSync with different threshold settings until a desirable conservation projection is reached. However, a 50 % cutoff threshold is a useful starting point. If the lower similarity threshold is set too high, too few sequences are selected, giving rise to too little evolutionary divergence, which results in the whole protein surface appearing as highly conserved. If the setting is set too low, structural homologs can be inappropriately selected, which has the effect of masking or washing out the detection of conservation hotspots (i.e. the whole protein surface appears non-conserved). To allow removal of redundant sequences from the final visualization, ProteoSync also allows filtering using an upper % identity threshold. Like the lower threshold, any hit sequences with % identity above the threshold are removed from the final alignment. This allows automated filtering of sequences that are extremely similar to the query and provide minimal diversity to the alignment.

There is also a possibility that a returned sequence is a small fragment compared to the full length of the query protein, which results in large swaths of the sequence being inappropriately marked as unconserved. For this reason, ProteoSync accepts a length variability threshold between 0 % and 100 % that filters sequences based on their length in relation to that of the query sequence. For example, if the threshold is set at 20 %, only those sequences whose length is between 80 % and 120 % of the query sequence’s length will be kept. Note that this parameter must also be tuned per protein, because it is possible that true orthologs may still be significantly shorter or longer than the query, particularly in large query proteins or those with long, unstructured loops. We found that a 20 % threshold was a useful starting point for smaller, compact proteins.

### 2.2 Aligning sequences and determining conservation with clustalw

ProteoSync then applies the CLUSTALW sequence alignment algorithm [11] to the predicted orthologous sequences to create a sequence alignment with conservation scores for each residue. The conservation scores are displayed using common alignment score conventions [12], where:

- ‘*’ indicates that the residue is identical across all species.
- ‘:’ indicates that there are conservative substitutions, meaning the residues are very similar across species.
- ‘.’ indicates that there are semi-conservative substitutions, meaning the residues are fairly similar across the aligned species.
- ‘ ‘ indicates that there are non-conservative substitutions, meaning the residues are not similar between the aligned species.

Also included is a conservation score based on the % of aligned sequences that perfectly match the query sequence. This score is represented as a string of Unicode characters at the bottom of the alignment, as follows:

- ‘ ‘ - 0–19 % of sequences match the query at that residue
- ‘-’ - 20–39 % of sequences match the query at that residue
- ‘━’ - 40–49 % of sequences match the query at that residue
- ‘▂’ - 50–59 % of sequences match the query at that residue
- ‘▃’ - 60–69 % of sequences match the query at that residue
- ‘▄’ - 70–79 % of sequences match the query at that residue
- ‘▅’ - 80–89 % of sequences match the query at that residue
- ‘▄’ - 90–99 % of sequences match the query at that residue
- ‘▆’ - 100 % of sequences match the query at that residue

### 2.3 Secondary structure analysis from PDB and AlphaFold structures

ProteoSync then uses the BLAST algorithm to search all protein sequences in the PDB (http://www.rcsb.org) for the closest sequence within a selectable identity threshold (same as that used for ortholog sequence search) for which an existing structure is available. A version of the PDB sequence database is provided in the software distribution package (ProteoSync/databases/pdb) and can be updated with the latest entries with the “update PDB database” button in the ProteoSync window. ProteoSync then downloads the PDB coordinate file (.pdb) for the best match and extracts the positions of helices and beta sheets in the protein using the DSSP algorithm [13]. ProteoSync then aligns the secondary structure elements to the sequence alignment as a string of characters, where:

- ‘-’ indicates that the residue is unstructured
- ‘A’ indicates that the residue is part of an alpha helix
- ‘B’ indicates that the residue is part of a beta strand
- ‘X’ indicates that the residue is not modelled in the pdb structure

For large proteins, multiple structures in the PDB may be identified corresponding to individual domains or segments rather than one structure for a whole protein. If the top hit from the PDB sequence search excludes large segments of the query sequence (20 residues or longer), the program runs iterative BLAST searches on the excluded regions to identify additional structure matches. All structures found are aligned on individual lines in the output alignment, labelled with their corresponding PDB codes. If the user entered a UniProt code [6], [7] for their protein, the program will also download the AlphaFold [14] predicted structure for the query protein (if available) and aligns its secondary structure to the sequence alignment in the same way as performed for the closest structure(s) in the PDB. The final multi-protein sequence alignment with conservation and secondary structure projection is then outputted into a text file**.** The output is stored in a new directory with the given run name created under the ‘Proteosync/outputs’ directory. Copies of any structure files analyzed during the process, either AlphaFold structures or PDB models, are included in the same output folder for easy reference. Run settings, including % length and identity threshold and the set of excluded species, as well as a list of accession codes for all sequences included in the alignment are included at the bottom of the file for easy referencing and validation.

### 2.4 PyMOL script generation for surface projection

Finally, PyMOL script files are created that allows projection of conservation from the alignment onto an empirically determined or predicted 3D model of the query protein downloaded by the user from the PDB (http://www.rcsb.org) or from the AlphaFold [14] repository (https://alphafold.ebi.ac.uk/).

Two script files are generated, which project distinct scoring schemes as coloring onto a protein’s surface. The first scheme corresponds to the discrete CLUSTALW alignment scores as follows:

- Dark blue indicates that the residue is identical across all species (equivalent to ‘*’ in the alignment)
- Medium blue indicates that there are conservative substitutions, meaning the residues are very similar across species (equivalent to ‘:’ in the alignment)
- Light blue indicates that there are semi-conservative substitutions, meaning the residues are fairly similar across the aligned species. (equivalent to ‘.’ in the alignment)
- Grey indicates that there are non-conservative substitutions, meaning the residues are not similar between the aligned species. (equivalent to ‘ ‘ in the alignment)

While blues are the default color shade, we include the option to select a range of other shades for the projection, including reds, greens, and grays. All shades share the convention in which darker colors correspond to higher conservation.

The second scoring scheme corresponds to the continuous value assigned to each residue representing the percentage of the aligned species that directly match the query sequence at that position. A higher percentage of matching sequences corresponds to a darker color. This color scheme is more robust to inclusion of one or more poor sequence matches in an ortholog search, as each single sequence has less impact on the overall score at a specific residue position. However, unlike the CLUSTALW scores, this scoring system does not take into account conservative substitutions (e.g. substituting a glutamic acid for an aspartic acid). As with the CLUSTALW scoring script, the % identity scoring script defaults to shades of blue but can be changed to other colours. The generated script files are stored in the same output folder created for the alignment.

## 3 Results

To demonstrate the utility of the program, we used ProteoSync to analyze 31 Fbox proteins containing Leucine Rich Repeat (LLR) or WD40 repeat domains. Fbox proteins are substrate selectivity subunits of the multi-subunit SKP1-cullin 1-F-box protein (SCF) type E3 ubiquitin ligases, which ubiquitinate target proteins, typically rendering them as substrates for degradation through the 26S proteasome [15], [16]. Each Fbox protein minimally possesses an Fbox domain, which binds to SKP1 to enable F-box protein recruitment into the SCF holoenzyme complex, and a WD40, LLR domain or other domain (for FBXW, FBXL and FBXO proteins, respectively), which typically function to recognize substrates. We focused our analysis on the FBXW and FBXL family members as the 41 FBXO family members display incredibly varied structures making comparison of results between family members difficult. While the substrate repertoires of many F-box proteins have been determined and visualized with atomic structures, a sizable fraction are poorly characterized, making the family an ideal test case for analysis by ProteoSync.

We started by analyzing the full suite of 31 selected Fbox proteins with an identity threshold of 50 % and a length threshold of 20 % or 50 %. We then repeatedly reanalyzed each protein, continuously lowering the % identity threshold until any/all identified conservation hotspots disappeared. We then kept the lowest % identity analysis for which there were characterizable conserved hotspots. We found that at too high % identity thresholds, most of the protein surface was labelled as conserved, and as the % identity was lowered, the regions of conservation receded until smaller hotspots remained.

**Fig. 2:**
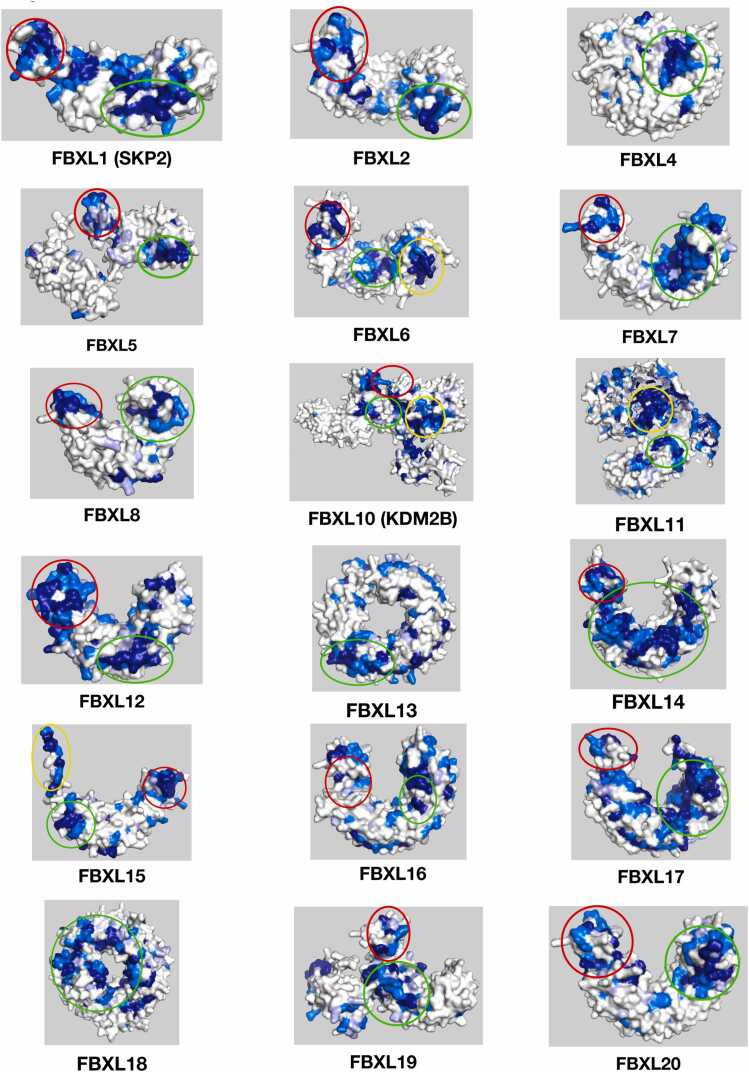
Snapshots of conservation hotspots identified by ProteoSync on 19 FBXL members of the Fbox family. Red circles highlight hotspots located on the Fbox domain, green circles highlight hotspots on the LRR domain, and yellow circles highlight hotspots on other domains.

**Fig. 3:**
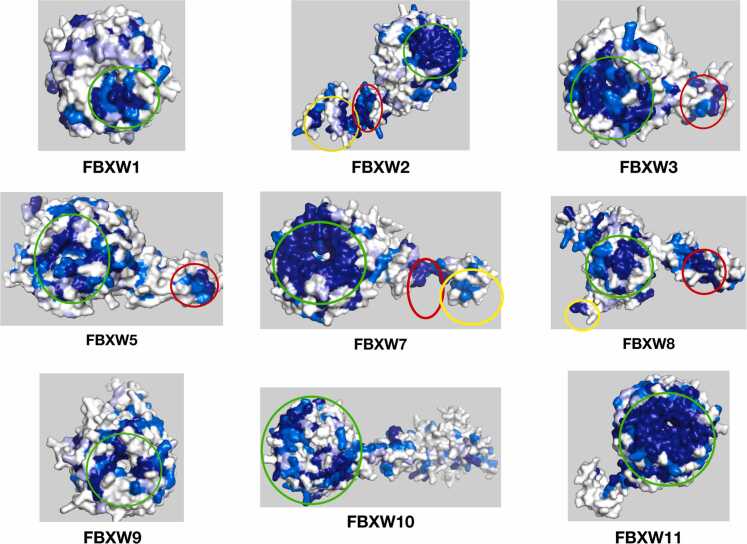
Snapshots of conservation hotspots identified by ProteoSync on 9 FBXW members of the Fbox family. Red circles highlight hotspots located on the Fbox domain, green circles highlight hotspots on the WD40 domain, and yellow circles highlight hotspots on other domains.

Out of the 31 proteins analyzed by ProteoSync, well defined hotspots of conservation were obtained for 28. Fig. 2, Fig. 3 show representative views for all 28 informative surface projections obtained. The majority of each protein’s surface is colored white, representing lack of conservation and thus an absence of inferable function, while clear conserved hot spots are visible elsewhere on the structure, suggesting sites of functional importance. Indeed, all 28 proteins feature a conserved surface corresponding to the shared Fbox domain that is expected to bind SKP1. Out of the 28 successful examples, 11 have structures reported in the PDB with biologically relevant ligands engaging the LRR or WD40 domain. When we align these structures with the conservation projections produced by ProteoSync, great agreement is observed between the predicted hotspots and the established ligand binding surfaces. Four Illustrative examples, namely FBXW7 and FBXL5, which have reported liganded structures, and FBXL4 and FBXW2, which don't, are reviewed below. The whole suite of test results is presented in Supplemental Information 3.

### 3.1 Analysis of FBXW7

**Fig. 4:**
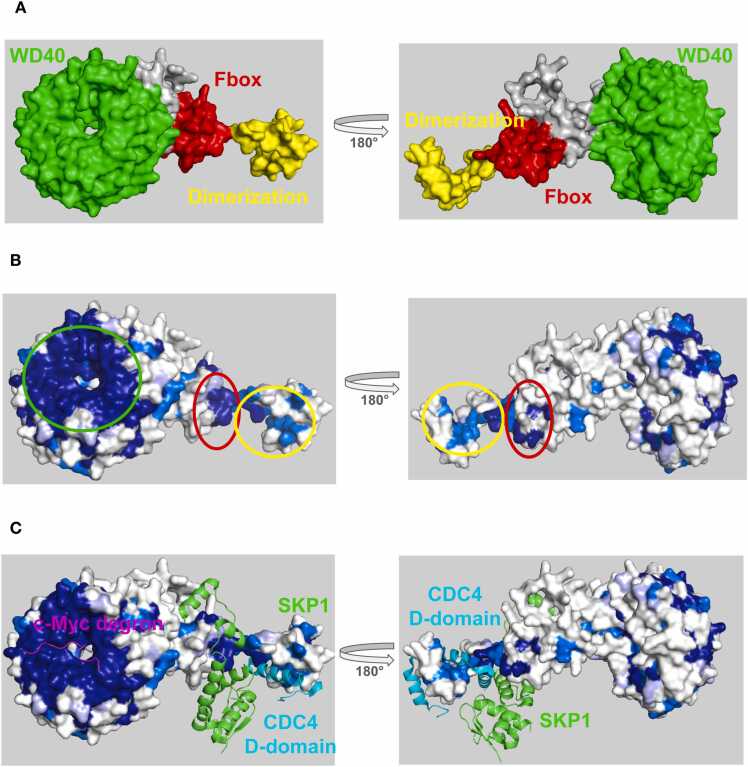
**A)** Structure of FBXW7 colored by domain. **B)** Conservation scores produced by ProteoSync projected onto the AlphaFold predicted structure of residues 222–707 of human FBXW7. Colouring is according to the CLUSTALW score generated in the output alignment (dark blue = invariant, medium blue = highly conserved with conservative substitutions, light blue = some conservation with semi-conservative substitutions, and white = not conserved). A hotspot of conservation on the front surface of the WD40 repeat domain identifies the functionally important degron binding pocket. **C)** Conservation hot spots of FBXW7 predicted by ProteoSync compared to experimentally determined binding surfaces from the Protein Database (PDB 7T1Z, 2P63). The predicted hotspots accurately correspond to the degron binding pocket on the WD40 domain and the SKP1 binding site located on the Fbox domain.

FBXW7 (a.k.a Cdc4 in the budding yeast) is responsible for binding mono or bi-phosphorylated peptide sequences termed degrons in a wide range of target proteins, rendering them substrates for ubiquitination and degradation [17], [18]. Well validated substrates include cyclin-E [19], the AP1 transcription factor c-JUN [20], and the intramembrane protease PS1 [21]. FBXW7 is composed of an N-terminal dimerization domain, a central Fbox domain, and a C-terminal WD40 domain (Fig. 4**a)**. ProteoSync analysis was performed using a 50 % identity threshold and 50 % length cutoff on the entire starter set of species and no intervention to remove faulty/truncated sequences from the resultant alignment (see Supplementary Information 4 for sequence alignment output). Projection of the calculated conservation scores onto the AlphaFold model of FBXW7 revealed a hot spot of invariant conservation on the front face of the WD40-repeat domain **(**Fig. 4**b)**, corresponding to the degron binding pocket [10], [22] **(**Fig. 4**c)**. Additional hot spots corresponding to the SKP1 binding site on the Fbox domain and a dimerization surface on the D-domain are also evident (Fig. 4**b,c**).

### 3.2 Analysis of FBXL5

**Fig. 5:**
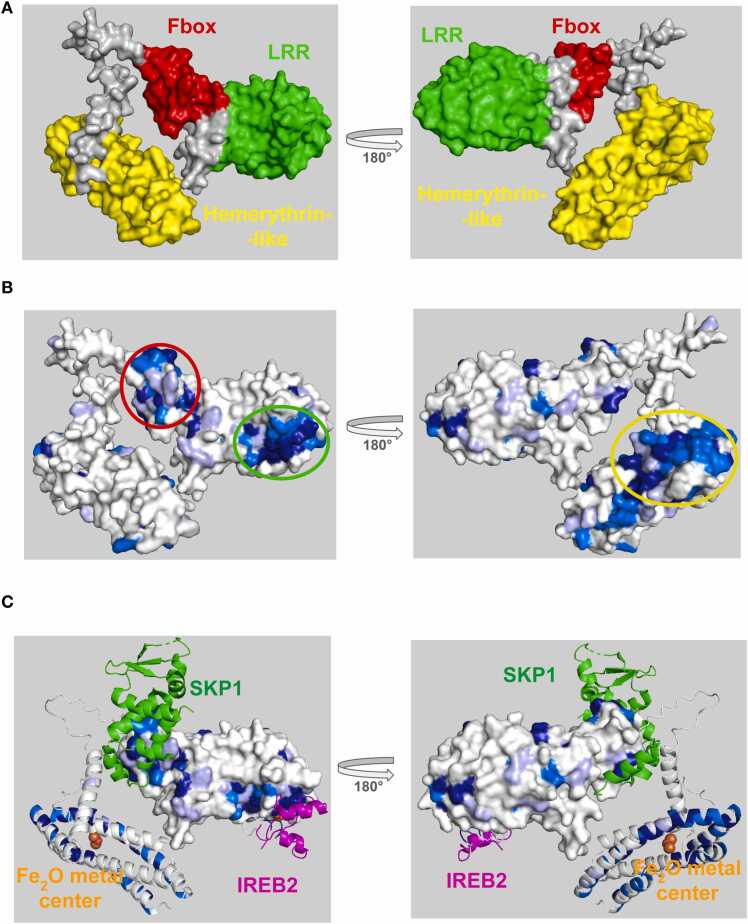
**A)** Structure of FBXL5 colored by domain. **B)** Conservation scores produced by ProteoSync projected onto the AlphaFold predicted structure of human FBXL5. Colouring is according to the CLUSTALW score generated in the output alignment as in Fig. 4. A hotspot of conservation is evident on the inner face of the LRR domain, where FBXL5 binds to the iron redox cluster. Another region of high conservation is located on the inner surfaces of the hemerythrin-like domain where iron is bound. **C)** Conservation hot spots of FBXL5 predicted by ProteoSync compared to experimentally determined binding surfaces from the Protein Database, including for Fe2S/IRP2 on the LRR domain (PDB 6VCD), SKP1 on the Fbox domain (PDB 6VCD) and for an Fe_2_O metal center on the hemerythrin-like domain (PDB 3V5Z).

FBXL5 is responsible for regulating iron homeostasis by controlling ubiquitination of iron-responsive element-binding protein 2 (IREB2) [23]. FBXL5 is composed of an N-terminal hemerythrin-like (Hr) domain, a central Fbox domain, and a C-terminal LRR domain (Fig. 5**a).** The LRR domain binds an iron redox cluster 2Fe-2S at high iron concentrations, which assists in the recruitment of IREB2 as a substrate for ubiquitination and subsequent degradation. Under high-iron conditions, the N-terminal hemerythrin-like (Hr) domain binds a diiron metal center, whereas in low-iron conditions, the absence of metal binding to the Hr domain causes FBXL5 to undergo a conformational change which leads to its eventual degradation [23], [24]. ProteoSync analysis was performed using a 40 % identity threshold and 20 % length cutoff on the entire starter set of species and no intervention to remove faulty/truncated sequences from the resultant alignment (see Supplementary Information 5 for sequence alignment output). Projection of the calculated conservation scores onto the AlphaFold model of FBXL5 revealed hot spots of conservation on the inner face of the LRR domain and on the inner surfaces of the hemerythrin-like domain **(**Fig. 5**b)** corresponding to the iron-binding and the IREB2 substrate binding surfaces revealed in CryoEM and X-ray co-crystal structures (PDB 6VCD, 3V5Z) [25], [26] **(**Fig. 5**c)**. An additional hot spot corresponding to the SKP1 binding site on the Fbox domain is also evident (**Figure 5bc**).

FBXL5 is an illustrative example where the recursive structure search was shown to be useful. There are two different crystal structures deposited in the PDB for FBXL5: the first structure includes a fragment of FBXL5 containing the Fbox and LRR domains in complex with SKP1 and IREB2. The second structure includes a fragment of FBXL5 containing the hemerythrin-like domain in complex with a di-iron metal center. For a complete representation of FBXL5’s secondary structure across all domain structures deposited in the PDB, both structures must be parsed. The initial PDB search returns the structure containing the Fbox and LRR domains, leaving the hemerythrin-like domain unmodelled. The recursive search then reruns the hemerythrin-like domain sequence, finding the second structure.

### 3.3 Analysis of FBXW2

**Fig. 6:**
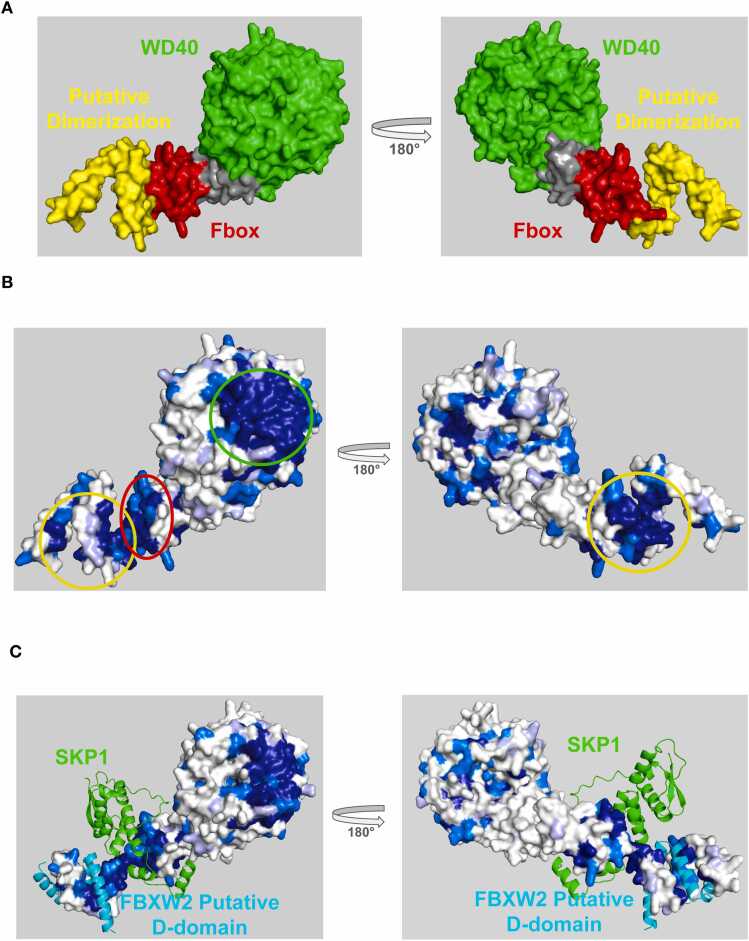
**A)** Structure of FBXW2 colored by domain. **B**) Conservation scores produced by ProteoSync projected onto the AlphaFold predicted structure of human FBXW2. Colouring as in Fig. 4. A hotspot of conservation is evident on the front face of the WD repeat domain, where FBXW2 is predicted to bind substrate. **C**) AlphaFold prediction of FBXW2 bound to SKP1 and to the dimerization domain of a second FBXW2 molecule.

FBXW2 is responsible for the ubiquitination and degradation of the epidermal growth factor receptor EGFR [27], Moesin [28], SKP2 [29], β-catenin [30] and GCMa [31]. Previous studies support a model whereby FBXW2 targets substrates through recognition of a phospho-degron motif with consensus TphosphoSXXXS/T, as identified in in EGFR, SKP2, and β-catenin [27], [29], [30]. At the time of publication, no structures of FBXW2 in complex with a substrate have been reported, and thus the precise degron binding site remained in question. FBXW2 is composed of N-terminal α-helical domain of unknown function, a central Fbox domain, and a C-terminal LRR domain (Fig. 6**a).** Here we use ProteoSync to predict a potential substrate binding site on the WD40 domain of FBXW2. ProteoSync analysis was performed using a 50 % identity threshold and 20 % length cutoff on the starter set of species except for the sea urchin *Lytechinus variegatus* and the hagfish *Eptatretus burgeri*, which were excluded from the search due to faulty/truncated sequences adversely affecting results (see Supplementary Information 6 for sequence alignment output). Projection of the calculated conservation scores onto the AlphaFold model of FBXW2 showed a highly conserved surface surrounding the front face of the WD40 domain **(**Fig. 6**b)**. Interestingly, this hotspot is rich in conserved arginine residues, a feature shared by two other FBOX proteins, FBXW7 and βTrCP, that also recognize phosphodegrons (of differing consensus) using conserved hotspots on their WD40 repeat domains [10], [22], [32]. A highly conserved surface is also apparent on the Fbox domain of FBXW2 implicated in SKP1 binding and on the unannotated N-terminal α-helical domain that AlphaFold predicts is a dimerization domain **(Figure 6bc).**

**Fig. 7:**
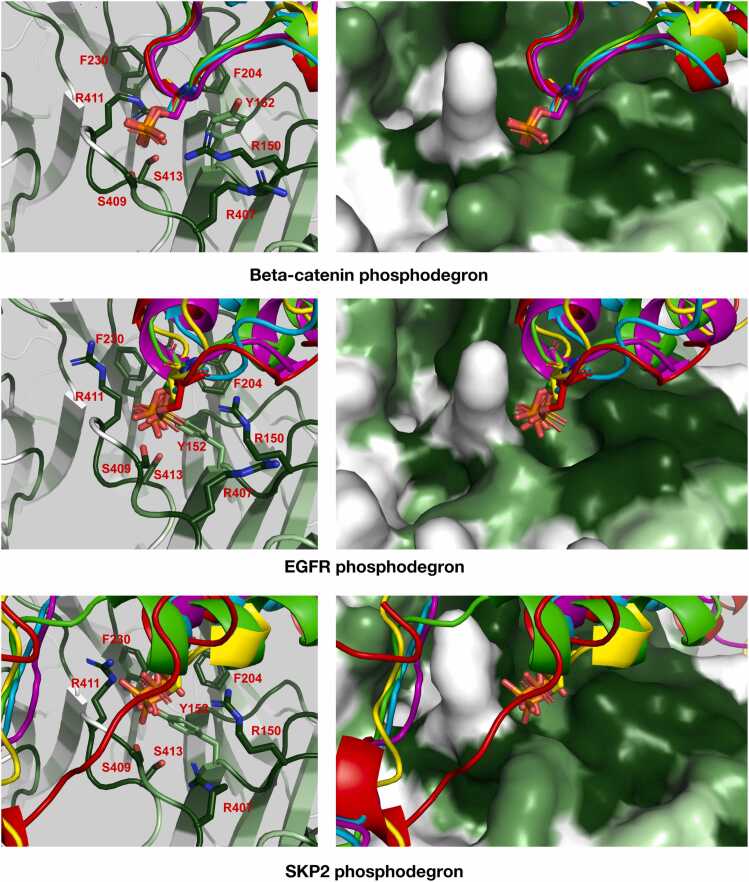
Superposition of AlphaFold predictions of FBXW2 binding to phosphodegrons of β-catenin (top), EGFR (middle) and SKP2 (bottom). Left and right views correspond to stick and surface representations of FBXW2, respectively. FBXW2 is colored according to conservation where darker shades of green correspond to higher levels of conservation (as in Fig. 4).

To investigate the potential role of the WD40 domain hotspot in substrate binding, we carried out AlphaFold predictions for FBXW2 with SKP2, β-catenin and EGFR. We ran AlphaFold on the full-length proteins (and also on 20 mer peptides encompassing their TSXXXS/T consensus motifs), with the first Ser position of the motif either phosphorylated or not phosphorylated. Intriguingly, the phosphorylated motif of each protein consistently engaged the hot spot on FBXW2 while the non-phosphorylated motifs did not (Fig. 7). In all cases, the phosphoserine was predicted to interact with a loop in the WD40 domain of FBXW2 (residues 406–416) containing several perfectly conserved arginine and serine residues.

### 3.4 Analysis of FBXL4

**Fig. 8:**
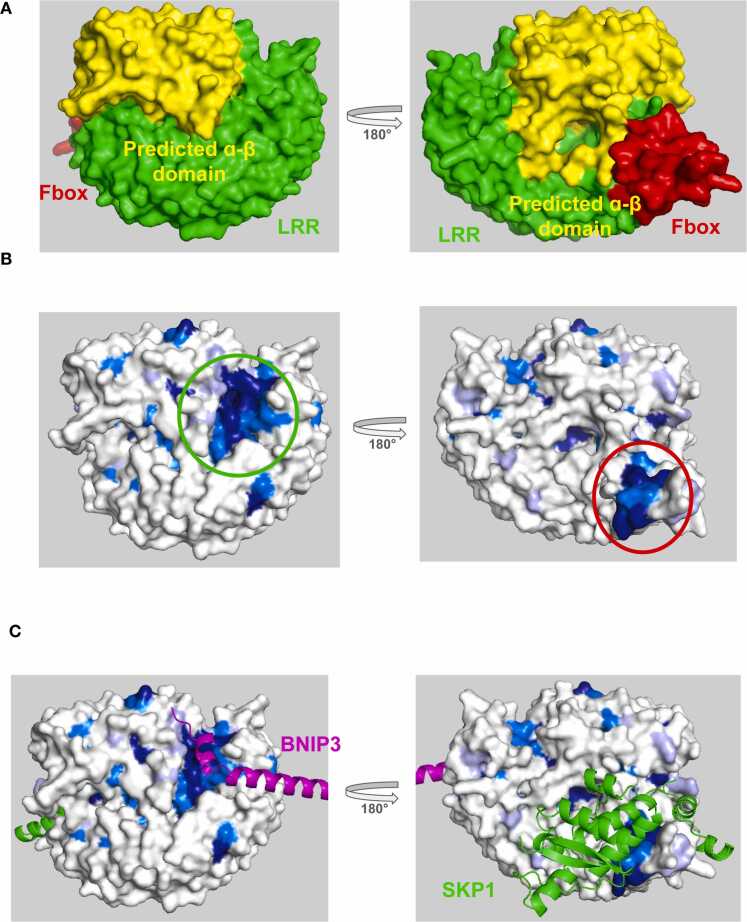
**A**) Structure of FBXL4 colored by domain. **B)** Conservation scores produced by ProteoSync projected onto the AlphaFold predicted structure of human FBXL4. Colouring as in Fig. 4. A hotspot of conservation is evident on the inner face of the LRR domain, where FBXL4 is predicted to bind substrate. **C**) AlphaFold structure prediction of FBXL4 bound to SKP1 and its substrate BNIP3.

FBXL4 is responsible for restraining mitophagy by regulating the ubiquitination and degradation of the mitophagy receptors BNIP3 and NIX [33]. Deletion analysis identified amino acid residues 151–184 in NIX and residues 161–225 in BNIP3 as required for ubiquitination by FBXL4 and subsequent degradation [34]. Whether the interaction between NIX and BNIP3 and FBXL4 is direct and where the reciprocal contact surface on FBXL4 that is responsible for substrate recruitment remains to be determined. At the time of publication, no structures have been reported for FBXL4 in complex with a substrate ligand. FBXL4 is composed of N-terminal domain predicted by AlphaFold to adopt a mixed α−β fold, a central Fbox domain, and a C-terminal LRR domain (Fig. 8**a).** Here we use ProteoSync to predict a potential substrate binding site on FBXL4. ProteoSync analysis was performed using a 40 % identity threshold and 20 % length cutoff on the entire starter set of species except for the annelid *Lamellibrachia satsuma* (see Supplementary Information 7 for sequence alignment output). Projection of the calculated conservation scores onto the AlphaFold model of FBXL4 revealed two hot spots of conservation **(**Fig. 8**b)**. The first is centered on the inner face of the LRR domain adjacent to the intramolecular binding site predicted by AlphaFold for the unannotated N-terminal domain. The second, is centered on the Fbox domain **(**Fig. 8**b)**. In contrast, the rest of the protein’s surface appeared almost entirely unconserved.

**Fig. 9:**
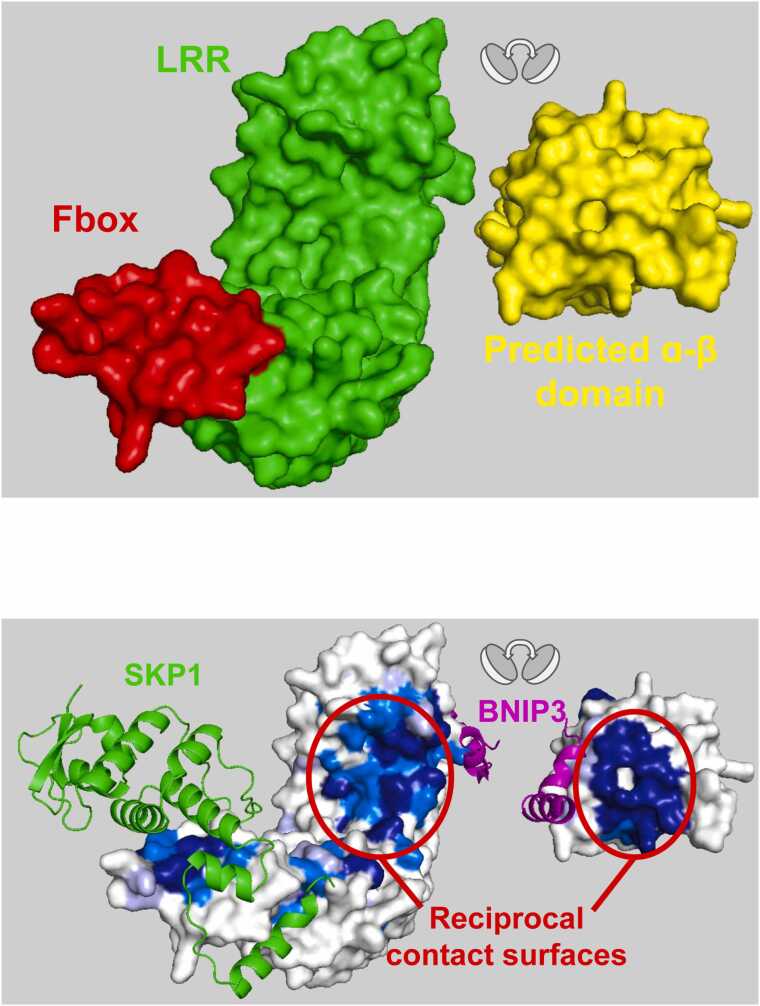
AlphaFold structure prediction of FBXL4 bound to SKP1 and BNIP3. The mixed α−β domain and LRR domain have been separated to reveal their intramolecular contact surfaces. Surfaces of FBXL4 are colored according to conservation as in Fig. 4, revealing well-conserved reciprocal contact surfaces.

To investigate the potential interaction function of the LRR domain hotspot of FBXL4 for substrate recognition, we carried out AlphaFold predictions for FBXL4 with the known substrates BNIP3 and NIX and the known canonical binding partner Skp1. As expected, Skp1 engaged the Fbox domain of FBXL4, while the proposed substrate BNIP3 engaged the exposed hotspot of conservation on the LRR domain (Fig. 8**c**). Interestingly, the intramolecular mode of interaction between the LRR domain and the novel N-terminal domain predicted by AlphaFold buries expansive surfaces that are identified by ProteoSync as highly conserved (Fig. 9). This finding supports the possibility that the predicted intramolecular binding mode between the LRR and mixed α−β domains is functionally relevant, and we speculate that it may represent a regulatory interaction that once relieved, exposes the two highly conserved buried surfaces for interaction with other proteins (putative regulators and substrates) in trans.

## 4 Discussion

ProteoSync is a program that can be used to quickly and easily calculate evolutionary conservation of a protein sequence across a user-defined set of species and project this conservation onto 3D atomic models visualized by PyMOL. Guided by visual inspection of the output, ProteoSync enables optimization of the CLUSTALW alignment to obtain a more meaningful surface projection. We have shown via analysis of members of the Fbox protein family that ProteoSync can accurately predict experimentally determined ligand interaction surfaces and predict previously uncharacterized interaction surfaces for many others. Occasionally, one or more problematic protein sequences is encountered in a search that lack a stretch of residues present in the bulk of other species sequences, potentially due to sequencing artifacts in the data obtained from NCBI or Ensembl. Due to the nature of the CLUSTALW algorithm, these missing regions cause a complete loss of detail in the conservation scores, such that the region is indiscriminately marked as unconserved. Problematic sequences bearing these deletions are easily spotted in the CLUSTALW output alignment file and can be removed via the taxonomy menu for the execution of a following ProteoSync analysis cycle.

As noted, there are several tools with functionality similar to ProteoSync, the most prominent of which is ConSurf [5]. ConSurf automates the same steps automated by ProteoSync and while excellent for most applications, several of its limitations are complemented by ProteoSync including:

a) Customizability: ProteoSync gives the user control over the set of species to be analyzed. The set of available species is easily extendible, and the user can import and curate custom datasets screened. The arrangement of the dataset subdirectories is automatically reflected in the species checkbox menu, allowing easy organization by phylogeny.
b) Integration of secondary structure information into the sequence alignment: This aspect allows easy comparison of output conservation scores with the known or predicted protein secondary structure based on related structures in the PDB or AlphaFold models.
c) Speed: ProteoSync runs very fast (seconds per run) compared to ConSurf, which can take hours for a single web server analysis.
d) Processing sequences in bulk: ProteoSync allows large groups of proteins to be analyzed in one submission, as showcased by our test case of 31 Fbox proteins.

The key advantages of ConSurf over ProteoSync include its more sophisticated method for selecting protein orthologs including removal of redundant sequences, and its method of assigning conservation scores based on the Rate4Site algorithm [35]. This may account for the slower run times compared to ProteoSync. In addition, the ConSurf web server features a convenient built-in viewing tool that displays the conservation projected onto a 3D model. Despite these differences, our test case analysis of 31 human Fbox proteins demonstrates that ProteoSync can successfully identify experimentally validated protein interaction surfaces and can predict potential protein interaction sites not previously characterized.

The advent of accurate structural prediction models, such as AlphaFold, has made prediction of functional hot spots more reliable for proteins that lack a solved crystal structure. Before the availability of AlphaFold, use of structure prediction/modeling methods (such as SWISS-MODEL [36]) could lead to inaccurate models where residues comprising a protein’s buried core instead face outwards towards solvent. Using such error prone models alongside programs such as ProteoSync would lead to highly misleading results, where artifact hotspots appear on the protein’s surface. With accurate structure models now accessible using AI based tools, these problems are greatly minimized. The following example illustrates this point. In prior work [18], we localized the phospho-degron binding pocket of yeast FBXW7 (aka Cdc4) using the process now automated by ProteoSync. Fortuitously, we succeeded, despite having modelled the WD40 domain of Cdc4 incorrectly with 7 propeller blades, when X-ray crystallography subsequently demonstrated that it consists of 8 propeller blades [22]. The eighth WD40 propeller blade eluded detection, but fortunately, did not contribute to the phospho-degron binding pocket. Building on the success with Cdc4/FBXW7, in unpublished work we attempted to map conservation hot spots in all 10 WD40 and all 21 LRR domain- containing Fbox proteins using homologous structures as modeling templates. Due to the imperfect modeling tools available at the time (we used SWISS-MODEL) and the fact that many of the LRR and WD40 repeat motifs in Fbox proteins are cryptically defined, the exercise proved highly uninformative. Errors in the modelled protein structures exposed hydrophobic core residues to solvent, manifesting as strong artifactual hotspots that masked the detection of functionally relevant hotspots. As shown in the current exercise, the availability of highly accurate AlphaFold remedied this problem.

ProteoSync has many practical applications including assisting in the engineering of optimal protein constructs for crystallization and for biochemical experimentation. Long, unstructured regions in a protein can inhibit protein crystallization and can lead to poor protein behavior in solution including aggregation and precipitation. While these unstructured segments can be readily identified in AlphaFold predicted models, conserved regions within flexible loops can contribute to protein folding or other conserved functions, which one may be keen to maintain. By projecting the conservation scores generated by ProteoSync onto a 3D structure model predicted by AlphaFold, a better picture can be obtained of what regions of a protein are likely dispensable or essential for a protein’s function. This represents another example of the utility of using ProteoSync in conjunction with AlphaFold.

Despite its utility, ProteoSync has limitations, including one related to the analysis of intrinsically disordered proteins (IDPs). Intrinsically disordered segments of proteins often show poor per-residue conservation across orthologs [37]. This makes analysis of sequence conservation more difficult. However, it is still possible to gather useful information about unstructured segments using ProteoSync either by running ProteoSync at a higher % identity threshold (to focus on the poorly conserved IDPs) combined with use of the CLUSTALW scoring scheme or by running ProteoSync at lower % identity threshold cutoff combined with the % identity-based coloring scheme. However, this methodology is not universally successful as the functional properties (such as conformational activity) of an IDP is often determined by coarse-grained sequence features, such as the quantity and distribution of positively and negatively charged residues, rather than by perfectly conserved individual residues [38]. Thus, any form of per-residue conservation analysis across orthologs often fails to perfectly capture all relevant information for IDPs. Other tools, such as CIDER [38], exist that analyze such sequence features to investigate an IDP’s conserved features.

The power of ProteoSync (and related tools such ConSurf and ProtSkin) lies foremost in the discovery of functional hotspots on globular domains, where conservation of residues is distributed sparsely and discontinuously along a protein’s primary structure, but projects to a focused hotspot in the context of the 3-dimensional globular structure. For IDPs, the protein fold has little, if any, bearing on finding a functional hotspot (until an IDP region engages a target protein and then becomes ordered). They are linear in nature and thus can be easily visualized using a linear sequence alignment tool (such as CLUSTALW) without need for projection on a 3D structure. If IDPs are of specific interest to a user, we recommend using programs specifically tailored for that purpose, such as CIDER, as mentioned above.

## CRediT authorship contribution statement

**Elliot Sicheri:** Writing – review & editing, Writing – original draft, Visualization, Software, Conceptualization. **Frank Sicheri:** Writing – review & editing, Writing – original draft, Supervision, Project administration, Funding acquisition, Conceptualization. **Daniel Mao:** Writing – review & editing, Writing – original draft, Supervision, Conceptualization. **Michael Tyers:** Writing – review & editing, Writing – original draft, Supervision, Project administration.

## Funding statement

Research was supported by the 10.13039/501100000024Canadian Institutes of Health Research (PJT-178026 to F.S., FDN-167277 to M.T.) and the 10.13039/501100004376Terry Fox Research Institute (TFRI 1107–04 to F.S.).

## Declaration of Competing Interest

The authors declare no conflicts of interest with respect to the research, authorship, and/or publication of this article.

## Footnotes

Appendix A Supplementary data associated with this article can be found in the online version at doi:10.1016/j.csbj.2025.09.012.
