# Supplementary material for: Analysis of Fbox substrate adapter proteins using ProteoSync, a program for projection of evolutionary conservation onto protein atomic coordinates
Source: Comput Struct Biotechnol J. 2025 Sep 11;27:4026–39. doi: 10.1016/j.csbj.2025.09.012 (PMC12475580; doi:10.1016/j.csbj.2025.09.012)
Supplement: Supplementary file 1 — Supplementary material [file mmc1.pdf]

Supplementary Information 1. Instructions for installing ProteoSync dependencies

ProteoSync requires the presence of several python packages to run. The can be installed by using pip with the included requirements.txt file. In the console, run:

- pip install -r /path/to/requirements.txt

ProteoSync calls on several external programs which need to be installed using the free program Anaconda.

First, install Conda, available at https://www.anaconda.com/products/distribution.

Second, open a terminal window and install the following packages using the command lines:

- conda install -c conda-forge dssp
- conda install -c bioconda clustalw
- conda install -c bioconda blast
