# Supplementary material for: Analysis of Fbox substrate adapter proteins using ProteoSync, a program for projection of evolutionary conservation onto protein atomic coordinates
Source: Comput Struct Biotechnol J. 2025 Sep 11;27:4026–39. doi: 10.1016/j.csbj.2025.09.012 (PMC12475580; doi:10.1016/j.csbj.2025.09.012)
Supplement: Supplementary file 2 — Supplementary material [file mmc2.pdf]

Supplementary Information 2. Instructions for synthesis of new species databases

Currently we have chosen a range of 84 species to include in our base installation, with a bias towards higher eukaryote diversity. There is a limited repertoire of bacterial, fungal and archaeal species. However, additional species can be added easily into the ProteoSync species dataset by following these instructions:

Step 1: Acquire a FASTA file containing all protein sequences of the species

We recommend acquiring a full annotated genome sequencing from Ensembl. If one is not available, a .fasta file containing all known sequences of a species's genome can be obtained from NCBI by following these instructions. If you already have a .fasta file for a species of interest, you can skip this step.

- Get the species' taxonomy id by searching for it at https://www.ncbi.nlm.nih.gov/Taxonomy/TaxIdentifier/tax_identifier.cgi
- On NCBI, select protein database and enter 'txid####[Organism:noexp]' (replace #### with the tax id)
    - We recommend also adding 'NOT partial' to exclude partial sequences from the results
- Near the top left of the search results, click 'Send to:', select File, set Format to FASTA, and click Create File

Step 2: Format the FASTA file into a BLAST database

- To format the database, you'll need the BLAST+ command line application. To install it, follow the instructions below.
    - Go to https://ftp.ncbi.nlm.nih.gov/blast/executables/LATEST/
    - Download ncbi-blast-2.14.0+.dmg
    - Open the installer and follow the instructions
- Create a new folder with the name of your species as you want it to appear on the taxonomy menu.
    - All spaces must be replaced by '_'.
    - Try not to use any other special characters.
- Change the name of your FASTA file from step 1 to the same name as the folder (except with .fasta at the end)
- Move the FASTA file into your new folder.

- Open terminal. Enter 'cd *[Insert path to your new folder here]*' to enter the correct directory
    - You can drag the folder into the terminal window and it will enter the path to it automatically.
- Type in 'makeblastdb -dbtype prot -in *[insert FASTA file name here]*'
- In the terminal it will tell you how many sequences were compiled into the database. Double check that this number is the **same as the number of search results from NCBI** when you downloaded the FASTA file.
    - The download occasionally terminates early and doesn't get every sequence, especially if you're downloading multiple FASTA files at the same time.
    - In the case that the numbers aren't the same, try downloading the FASTA file again and repeat step 2.
- There should now be 3 new files in the folder, ending in .phr, .pin, and .psq
- You can remove the original FASTA file if you want to reduce storage consumption. Only the 3 newly generated files are necessary.
- You now have a properly formatted BLAST database.

Step 3: Insert the new folder into the database file tree

- In the ProteoSync folder, there's a folder called databases. In this folder is another folder called species, which contains all the species-specific databases.
- The directories in the species folder are organized in a tree structure by taxonomy. The GUI uses this file structure to generate the taxonomy settings window.
- Insert your new folder into the file tree where it belongs taxonomically. Your species should then appear on the taxonomy menu the next time you open the application.
- NOTE: You can expand the taxonomic tree structure to include more taxonomic groups by creating new directories to organize your species folders. These groups will also appear on the taxonomy settings menu.
    - Make sure folder names don't contain spaces or unnecessary special characters
    - Make sure that individual species database folders contain **no other directories**. If a directory contains any subdirectories, it is assumed to be a taxonomic group folder, and the species database will not be found.
