# Supplementary material for: Analysis of Fbox substrate adapter proteins using ProteoSync, a program for projection of evolutionary conservation onto protein atomic coordinates
Source: Comput Struct Biotechnol J. 2025 Sep 11;27:4026–39. doi: 10.1016/j.csbj.2025.09.012 (PMC12475580; doi:10.1016/j.csbj.2025.09.012)

Supplementary Information 3. Results of ProteoSync analyses on the suite of FBXL and FBXW proteins

This document contains figures demonstrating the results of our analysis on the full range of FBXL and FBXW proteins.

Identified hotspots will be highlighted with colored circles. Text boxes of the corresponding color give details about the location of the corresponding hotspot.

For proteins with solved structures in the PDB, figures are included showing the projected alignment scores from ProteoSync overlaid onto those solved structures. The interaction areas are highlighted by colored circles, using the same color scheme to identify the corresponding conserved hotspots.

FBXL Hotspots

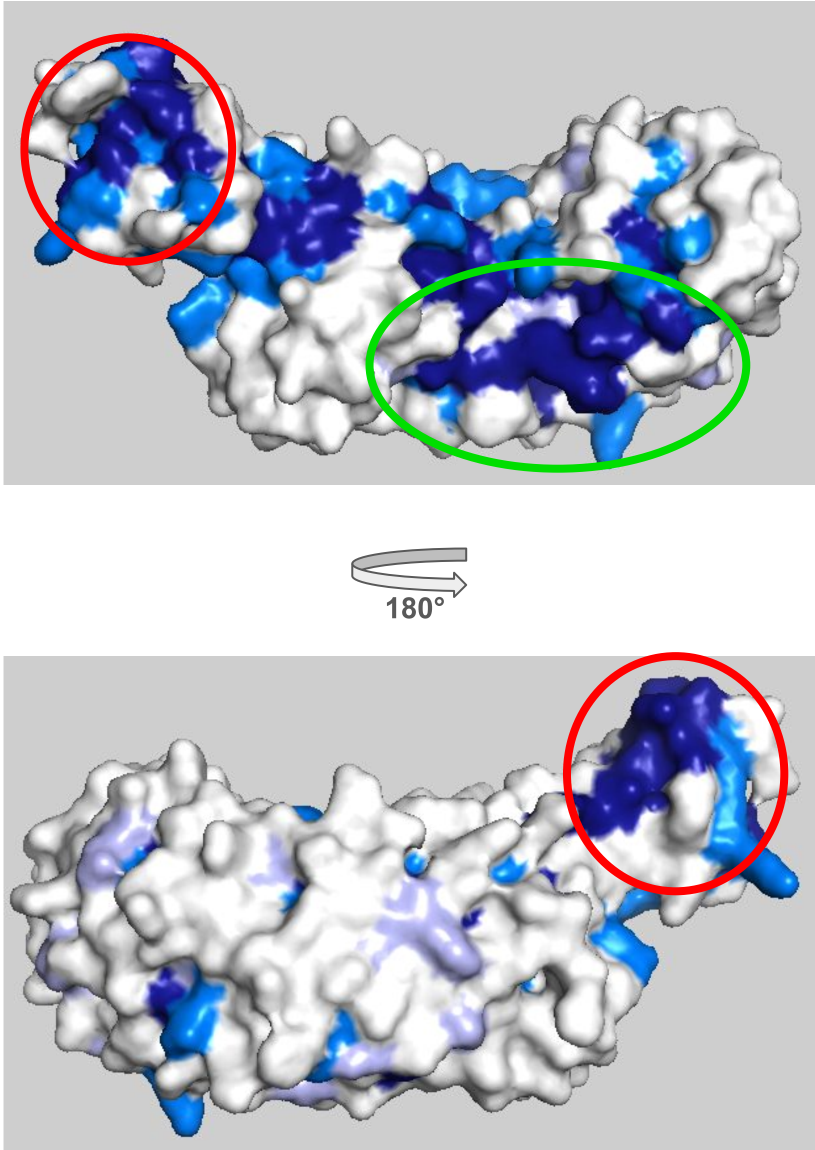

FBXL1 (SKP2)

Run settings:

- % identity threshold: 40%
- % length variability threshold: 50%

Well conserved surface covering Fbox domain

Well conserved region on interior face of LRR domain

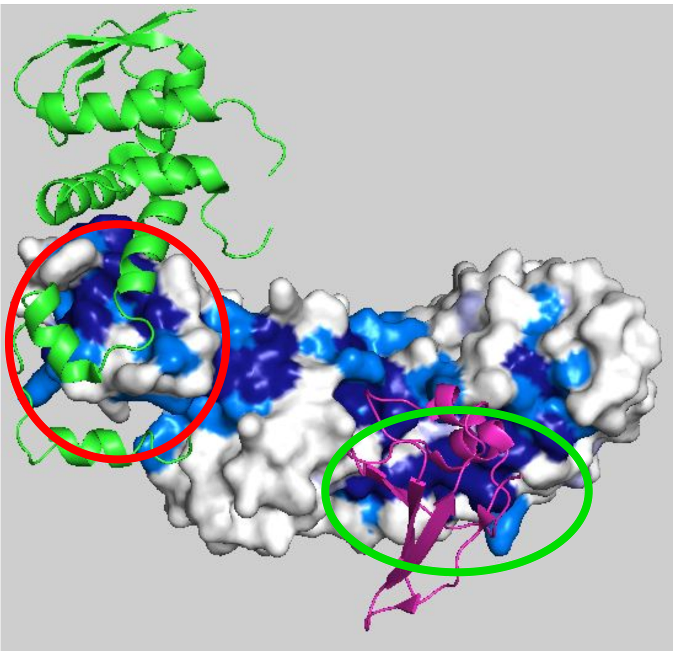

FBXL1 – PDB 2ASS

Green: SKP1
Magenta: CKS1

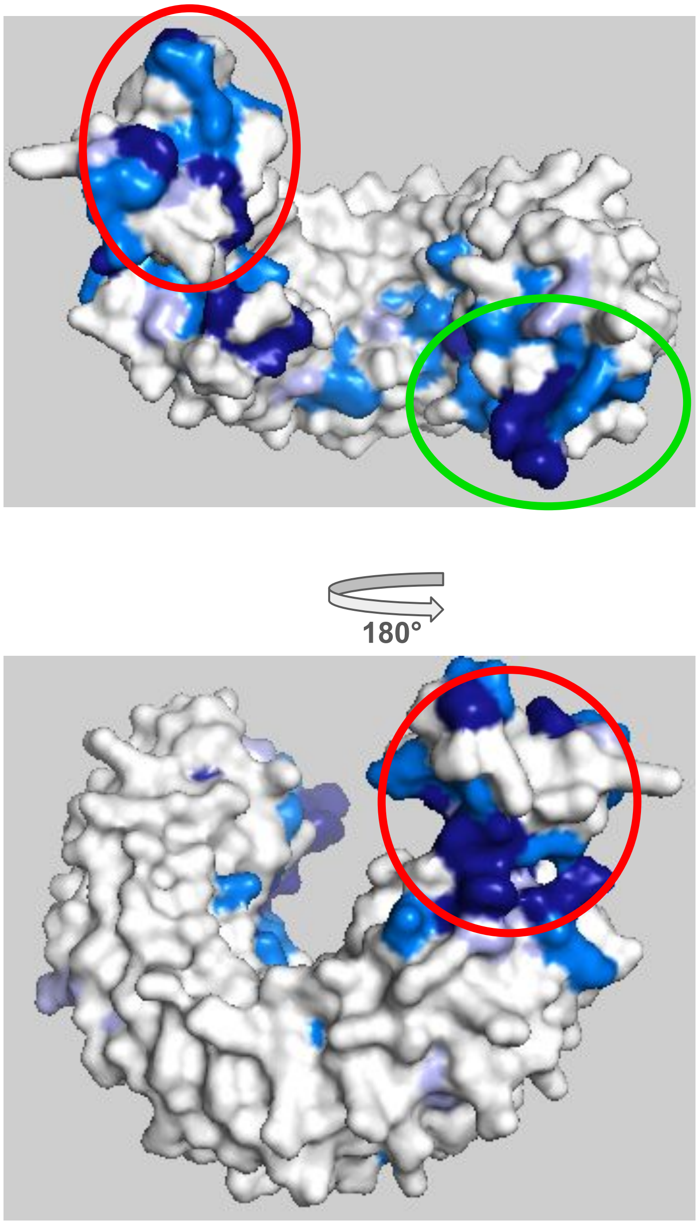

FBXL2

Run settings:

- % identity threshold: 40%
- % length variability threshold: 20%

Well conserved surface covering Fbox domain

Well conserved patch located on outer edge of the LRR domain

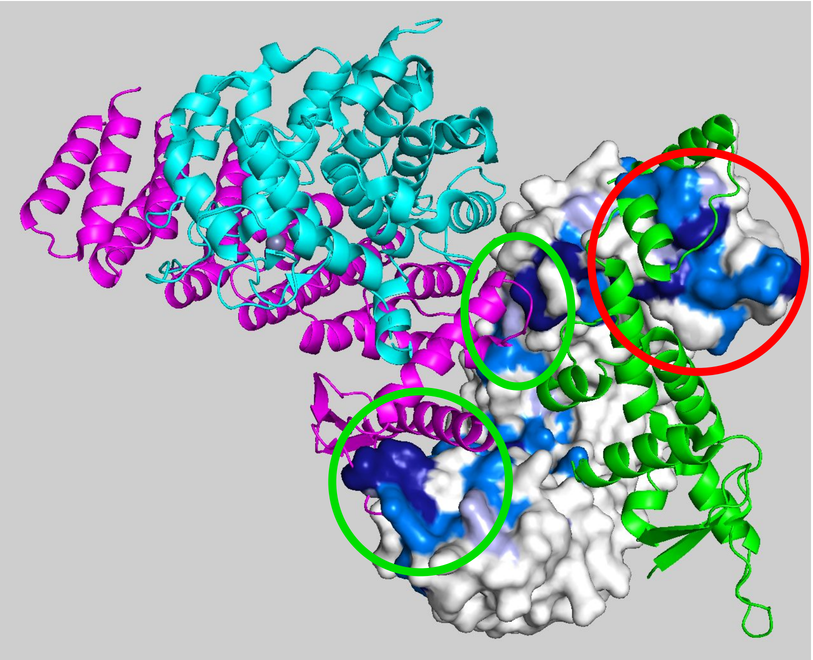

FBXL2 – PDB 6O60

Green: SKP1
Magenta: PTAR1
Cyan: GGTB

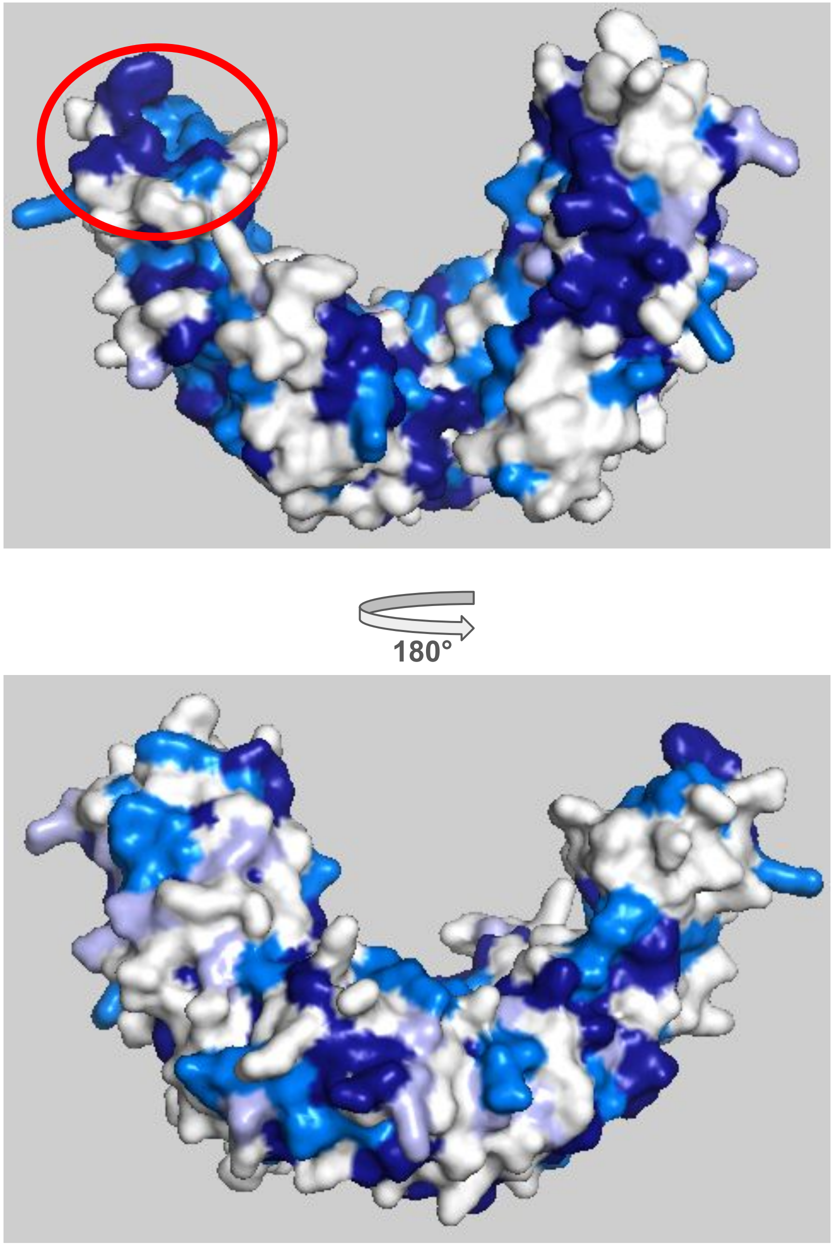

FBXL3

Run settings:

- % identity threshold: 40%
- % length variability threshold: 50%

Conserved patch on Fbox domain

No other significant hotspots located. Mottled conservation present across whole protein surface

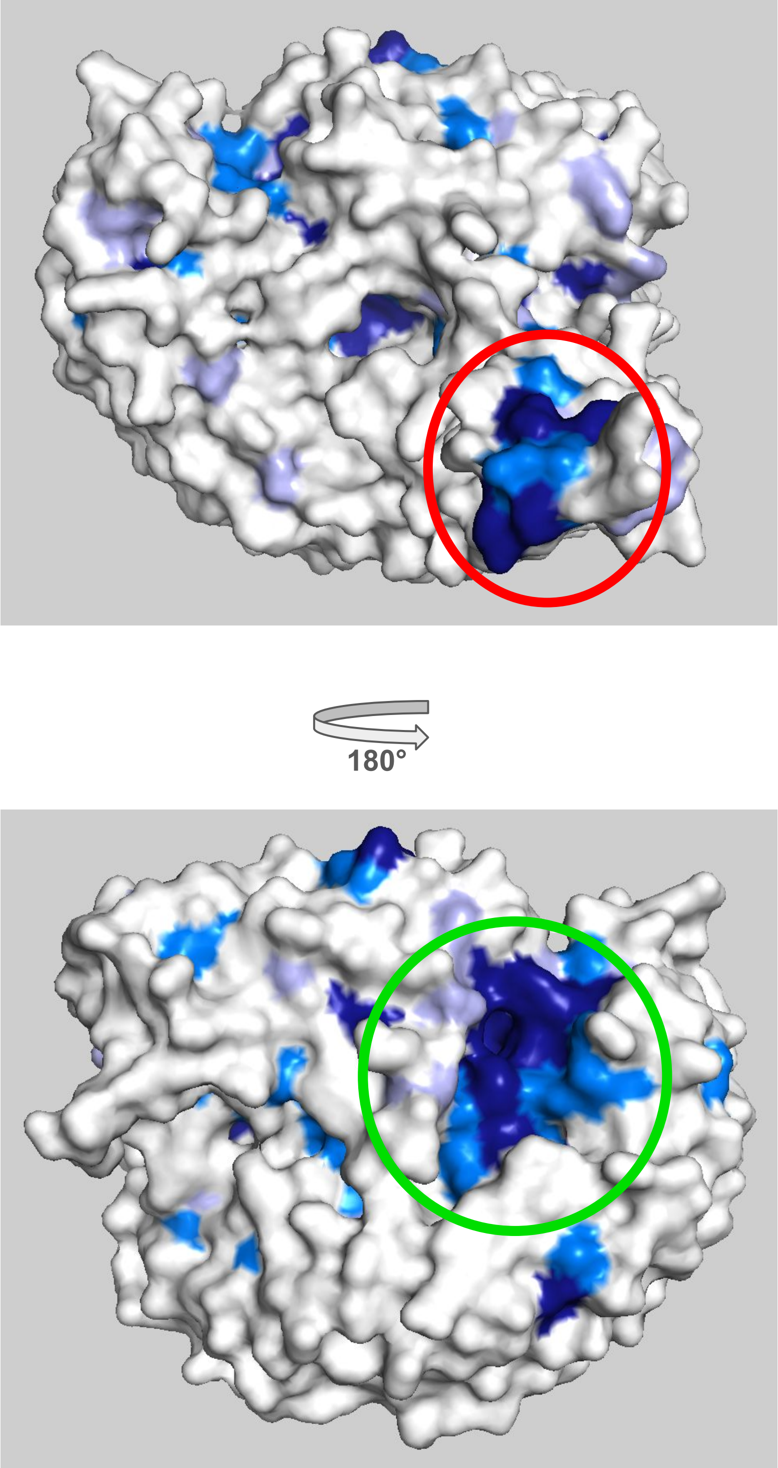

FBXL4

Run settings:

- % identity threshold: 40%
- % length variability threshold: 20%
- Removed species: Lamellibrachia satsuma, Nematostella vectensis

Small, well conserved patch located on exterior face of the Fbox domain

Large, well conserved patch located on interior faces between LRR domain and unnamed domain composed of 2 beta sheets (aa 49-230)

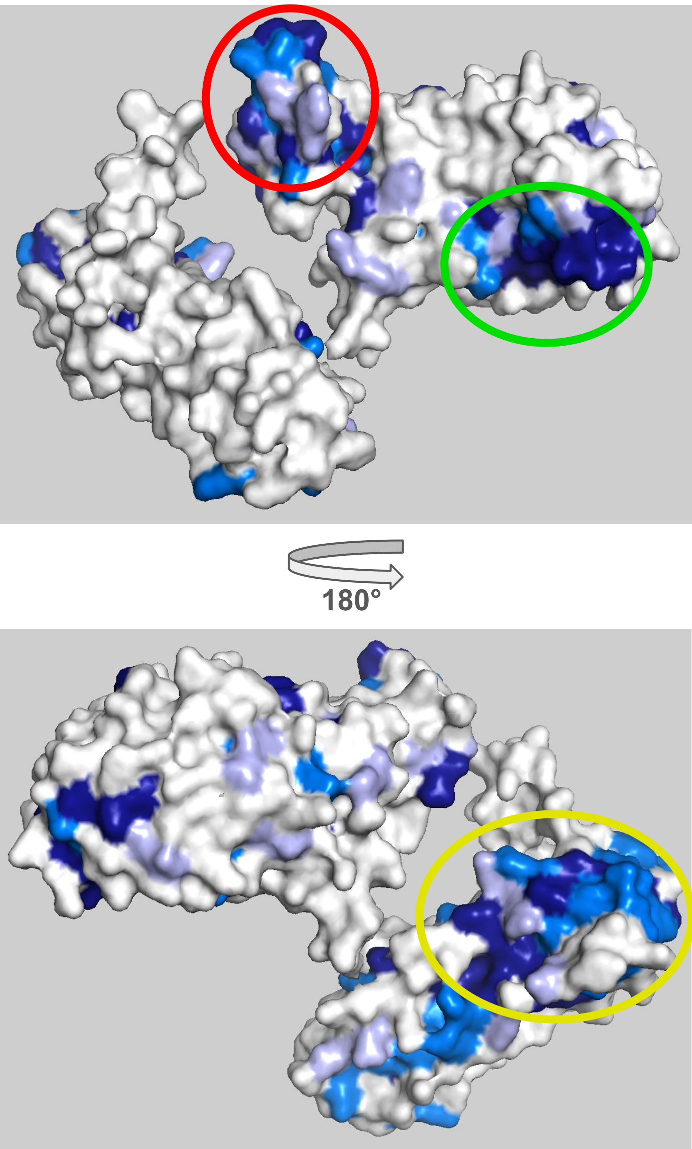

FBXL5

Run settings:

- % identity threshold: 40%
- % length variability threshold: 20%

Small, well conserved patch located on exterior face of the Fbox domain

Perfectly conserved patch located on interior face of LRR domain

Large, well conserved surface located on interior face of Hemerythrin-like domain

FBXL5 – PDB 6VCD (left), PDB 3V5Z (right)

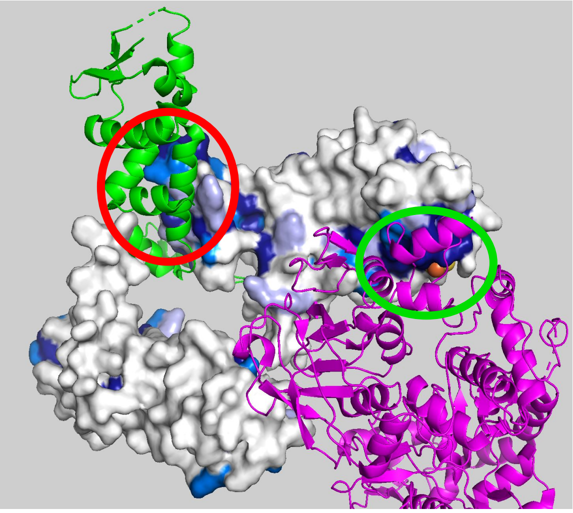

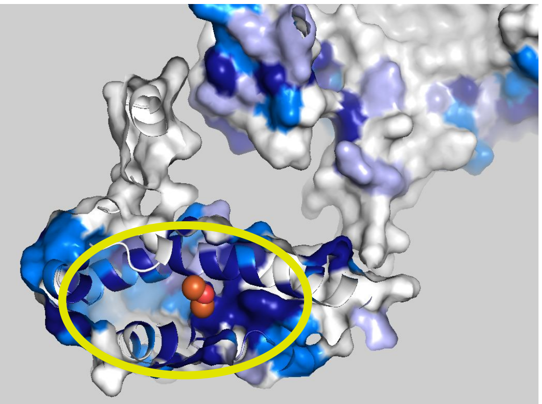

Green: SKP1
Magenta: IREB2
Orange/**Yellow** Molecule: Fe_2S_2
Orange/**Red** Molecule: Fe_2O

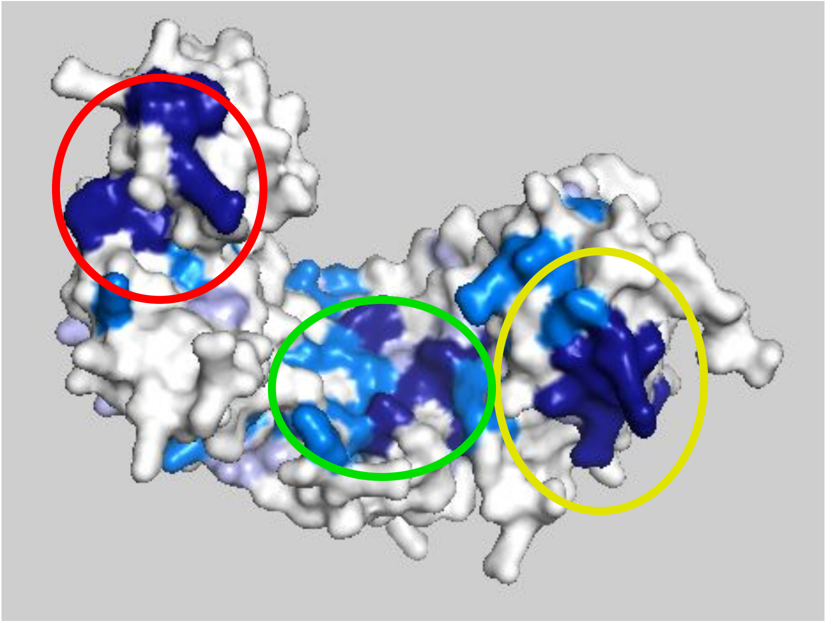

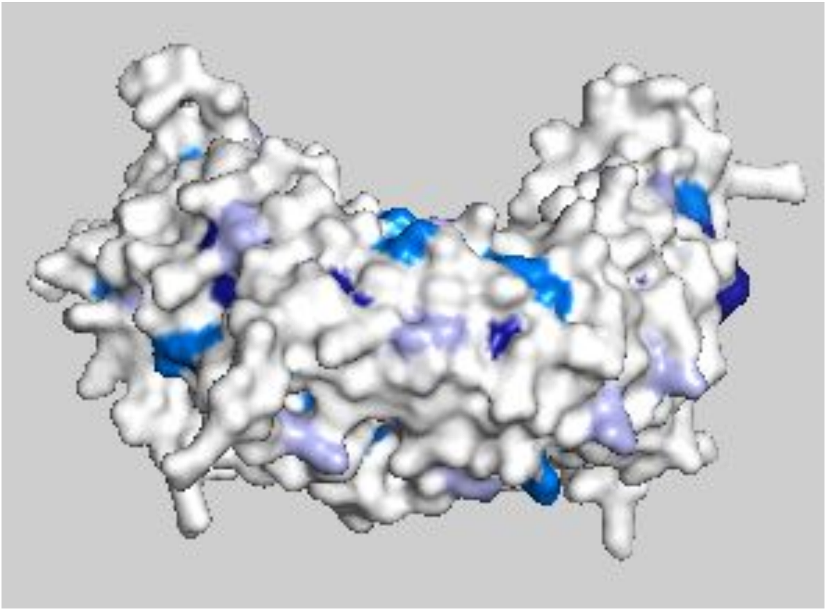

FBXL6

Run settings:

- % identity threshold: 40%
- % length variability threshold: 50%

Small, well conserved patch located on exterior face of the Fbox domain

Well conserved surface located on interior surface of LRR domain

Small, well conserved patch located on outer end of LRR domain

FBXL7

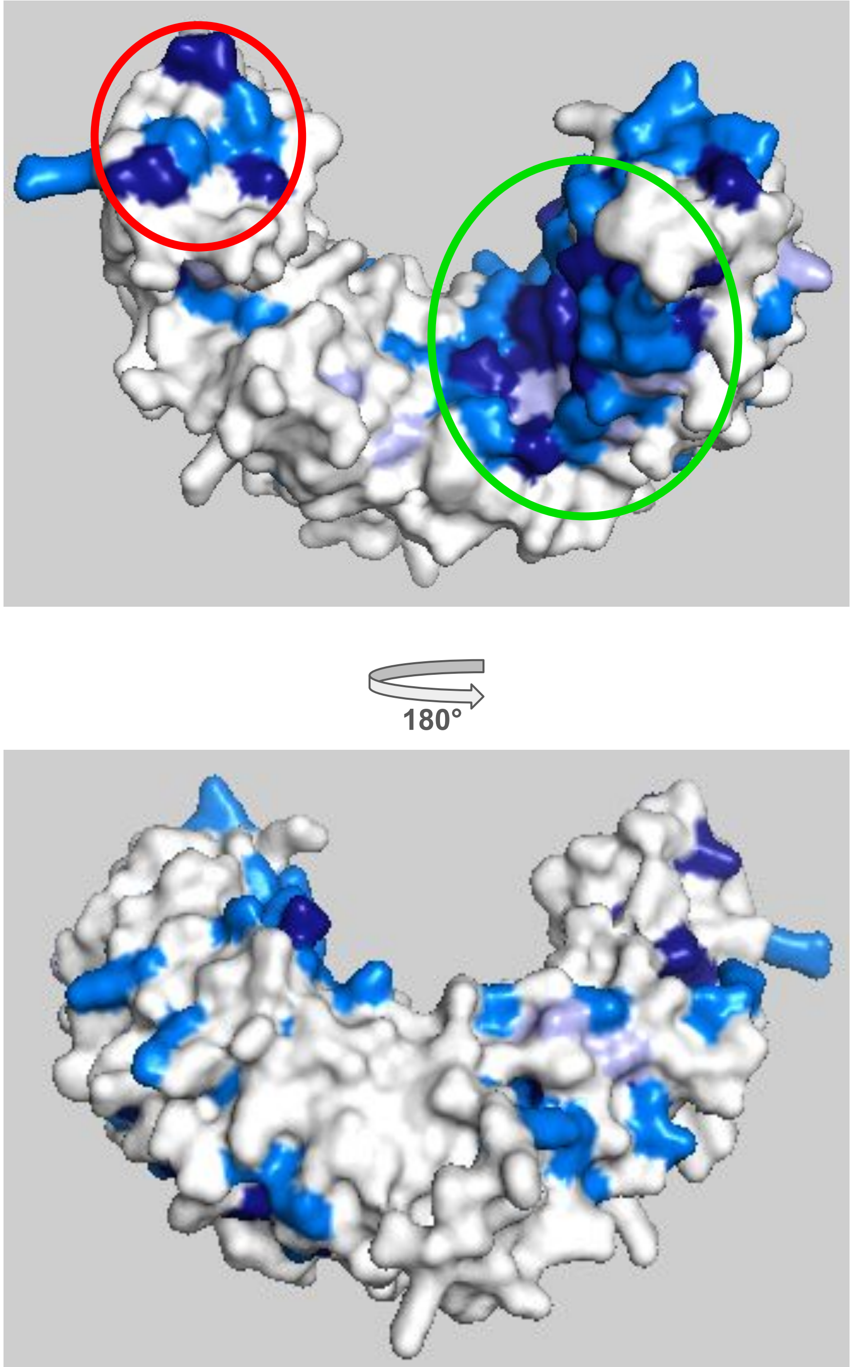

Run settings:

- % identity threshold: 40%
- % length variability threshold: 20%

Small, well conserved patch located on exterior face of the Fbox domain

Large, well conserved surface located on interior surface of LRR domain

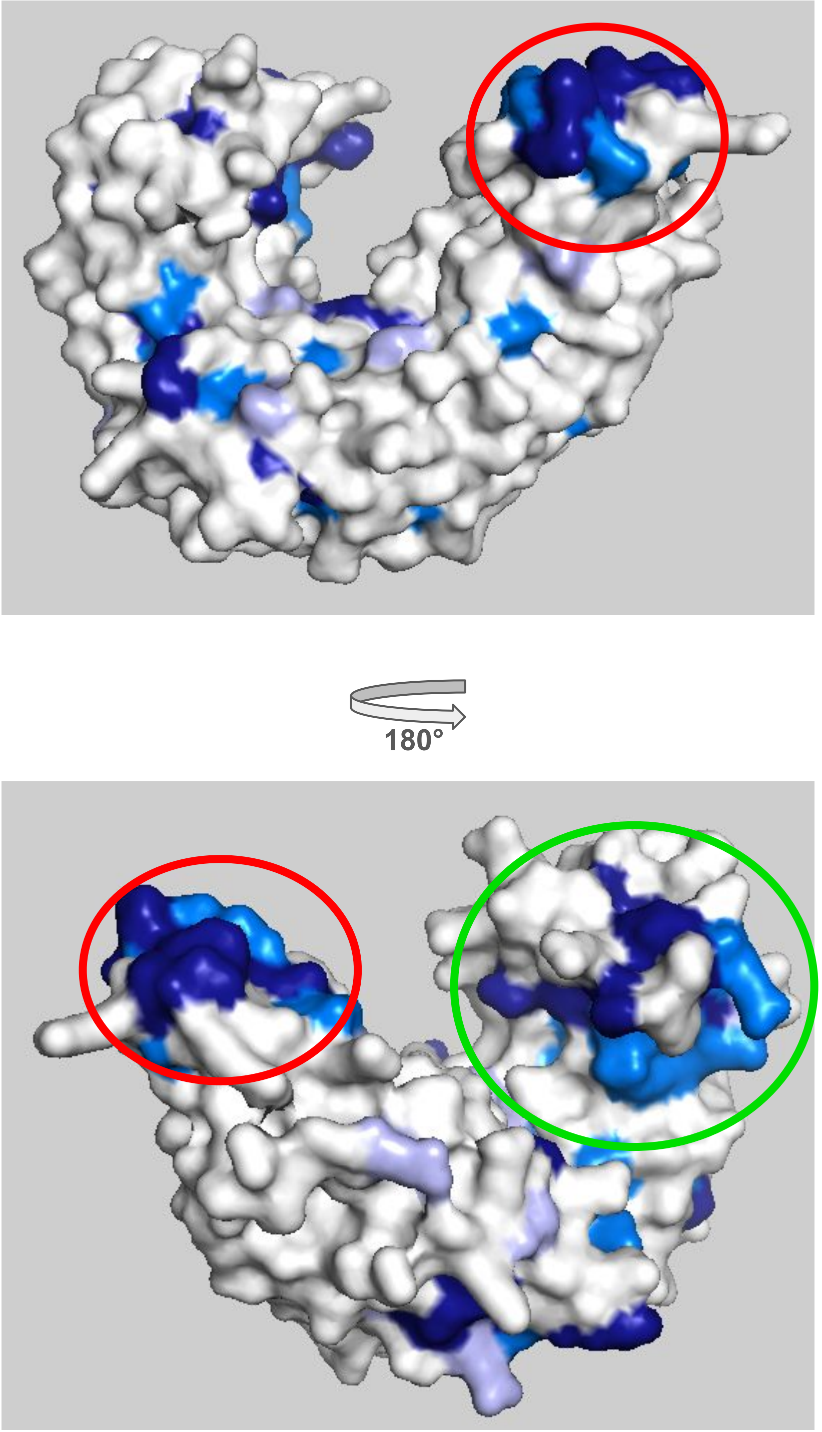

FBXL8

Run settings:

- % identity threshold: 40%
- % length variability threshold: 20%

Small, well conserved patch located on exterior face of the Fbox domain

Well conserved patch located on end of LRR domain

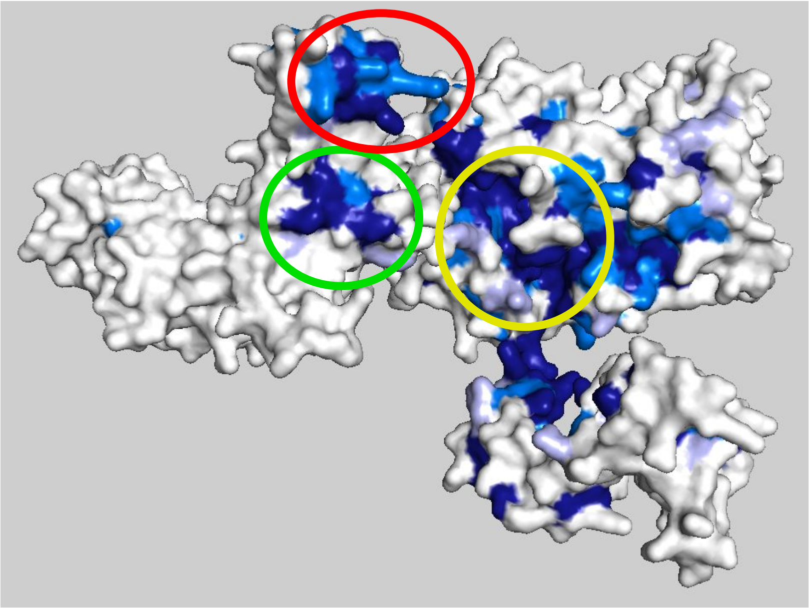

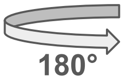

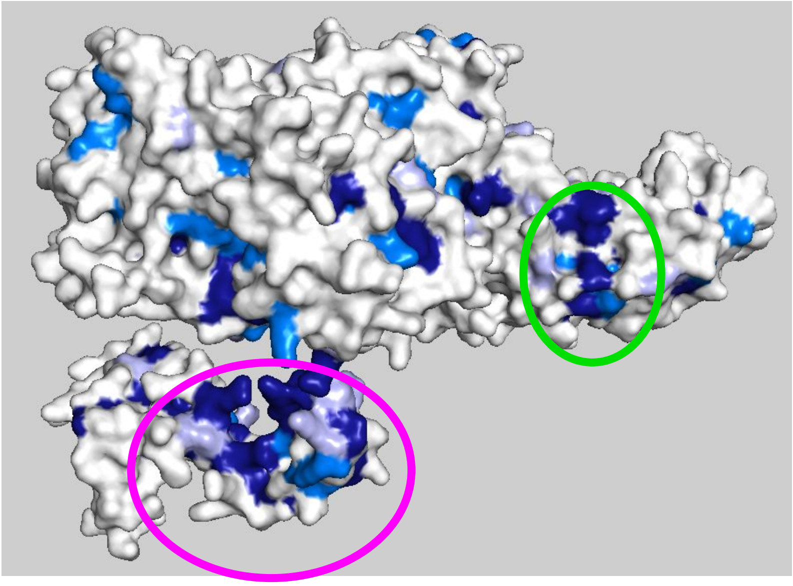

FBXL10 (KDM2B)

Run settings:

- % identity threshold: 30%
- % length variability threshold: 30%
- Excluded species: Otolemur garnetti

Small, well conserved patch located on exterior face of the Fbox domain

Two well conserved patches on either side of the LRR domain

Large, perfectly conserved surface located on the interior of the beta sheet barrel of the JmjC domain

Well conserved patch located in cleft of the CXXC-type zinc finger domain

FBXL10 Continued

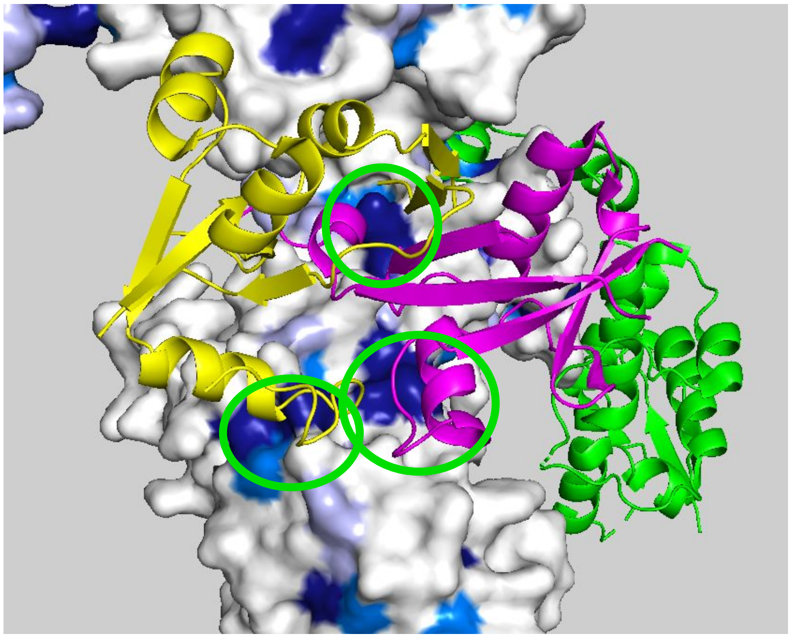

FBXL10 (KDM2B) – PDB 5JH5

Green: SKP1
Magenta: PCGF1 (Polycomb group RING finger protein 1)
Yellow: BCORL1 (BCL-6 corepressor-like protein 1)

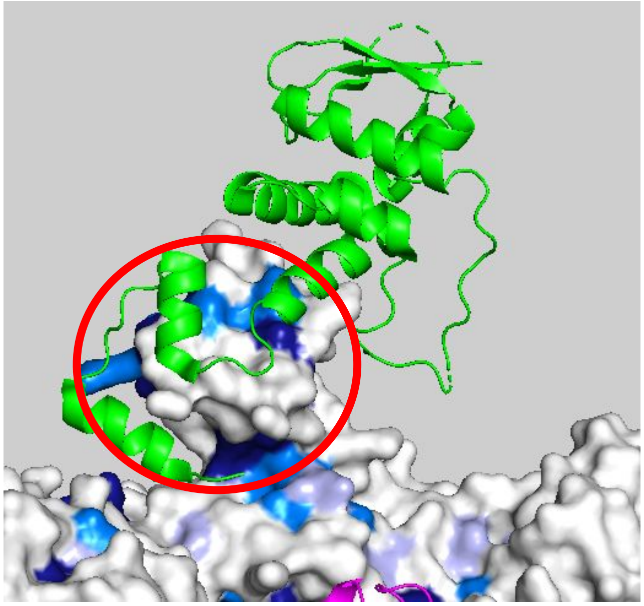

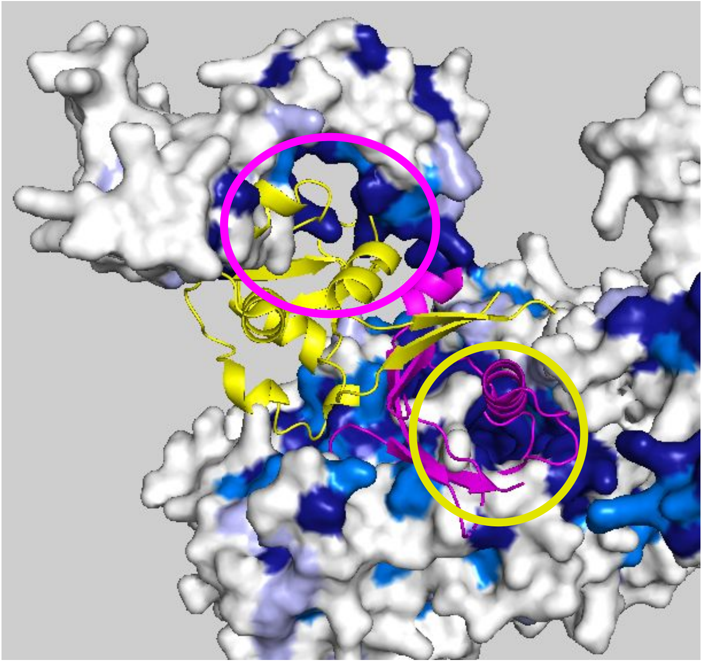

FBXL10 (KDM2B) – PDB 8HCU

Magenta: PCGF1 (Polycomb group RING finger protein 1)
Yellow: BCOR (BCL-6 corepressor)

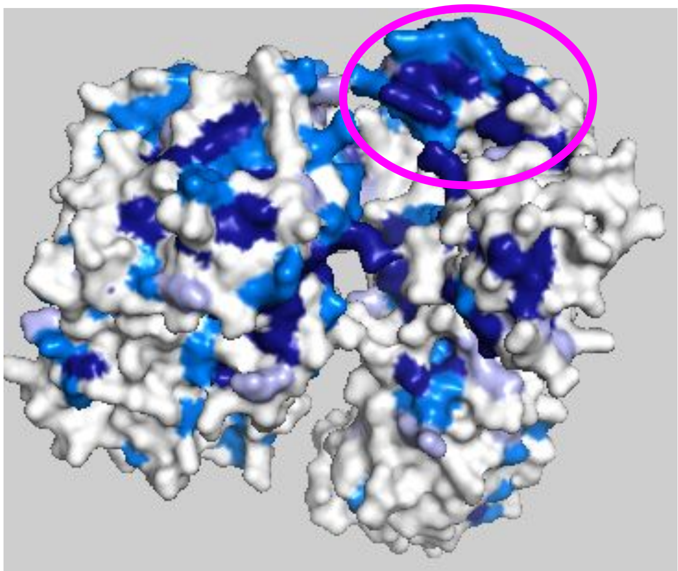

FBXL11

Run settings:

- % identity threshold: 40%
- % length variability threshold: 20%

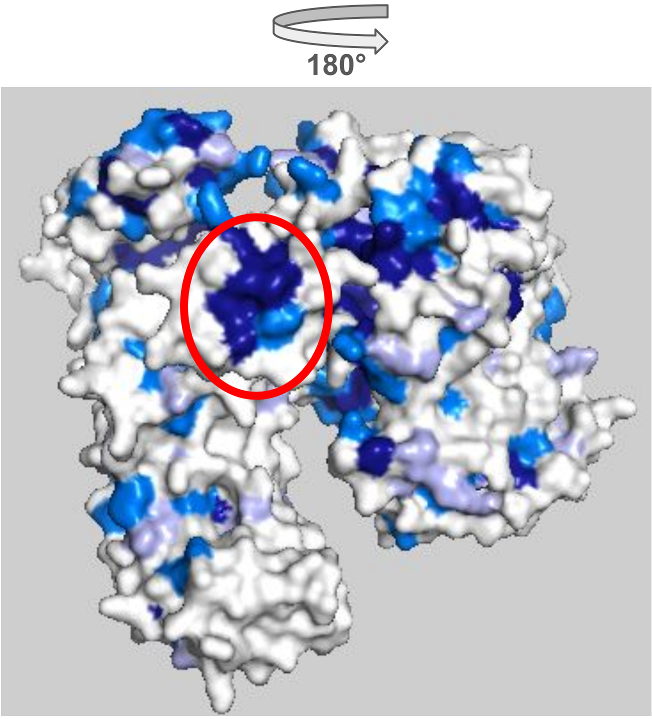

Small, well conserved patch located on exterior face of the Fbox domain

Small, well conserved patch located on surface of the LRR domain directly facing the opening of the beta sheet barrel of the JmjC domain

Large, perfectly conserved surface located on the opening and interior of the beta sheet barrel of the JmjC domain

Well conserved patch located in cleft of the CXXC-type zinc finger domain

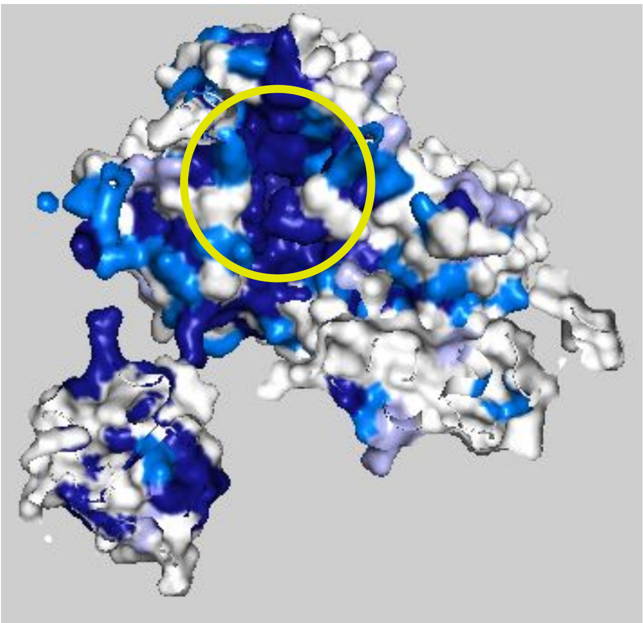

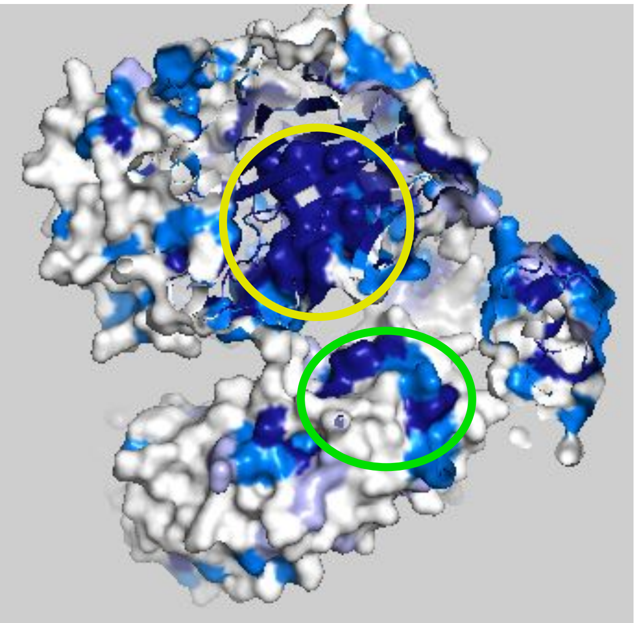

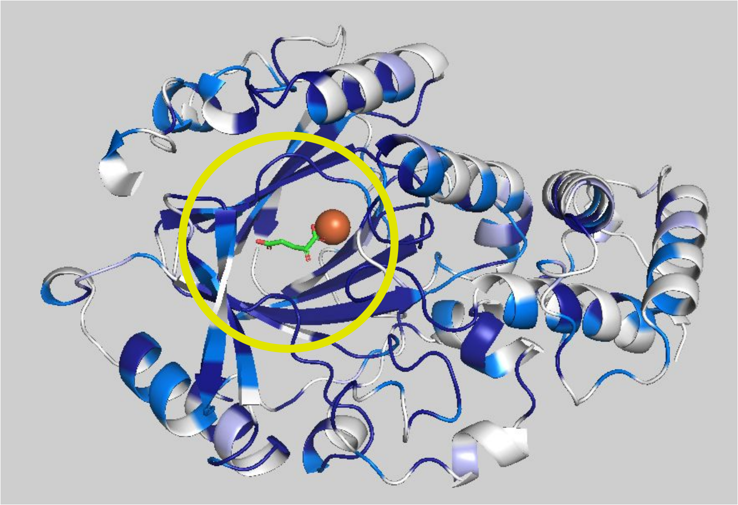

FBXL11 Continued

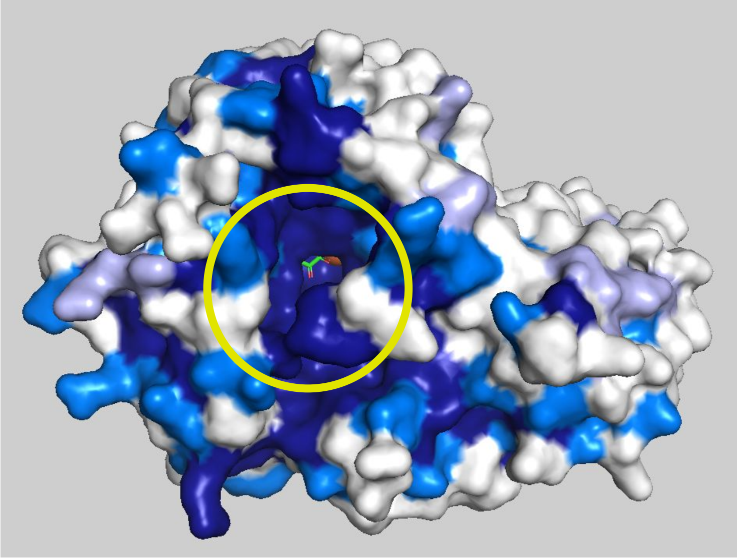

FBXL11 – PDB 2YU1

Green molecule: 2-oxoglutaric acid ($C_5H_6O_5$)
Orange ion: Fe^{2+}

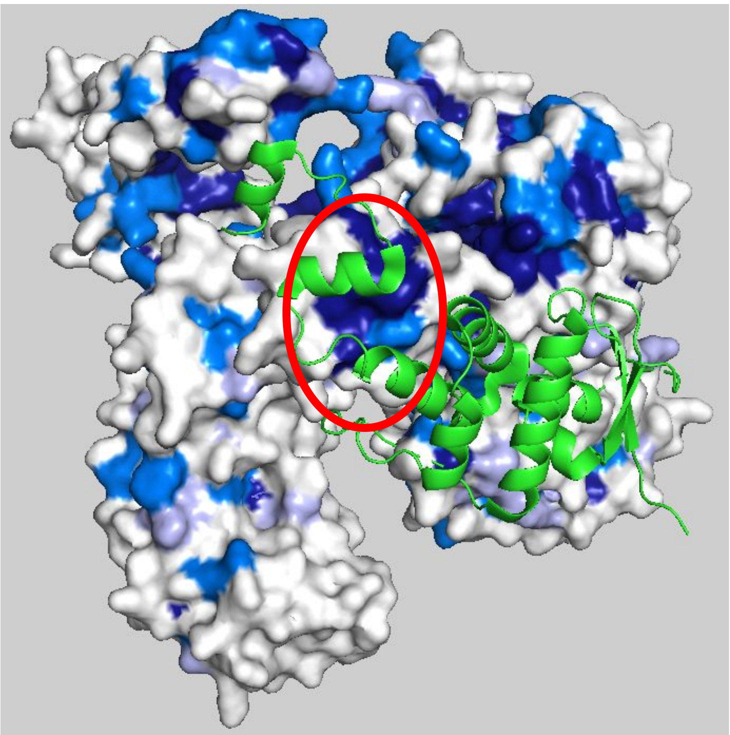

FBXL11 – PDB 6BYH

Green: SKP1

FBXL11 Continued

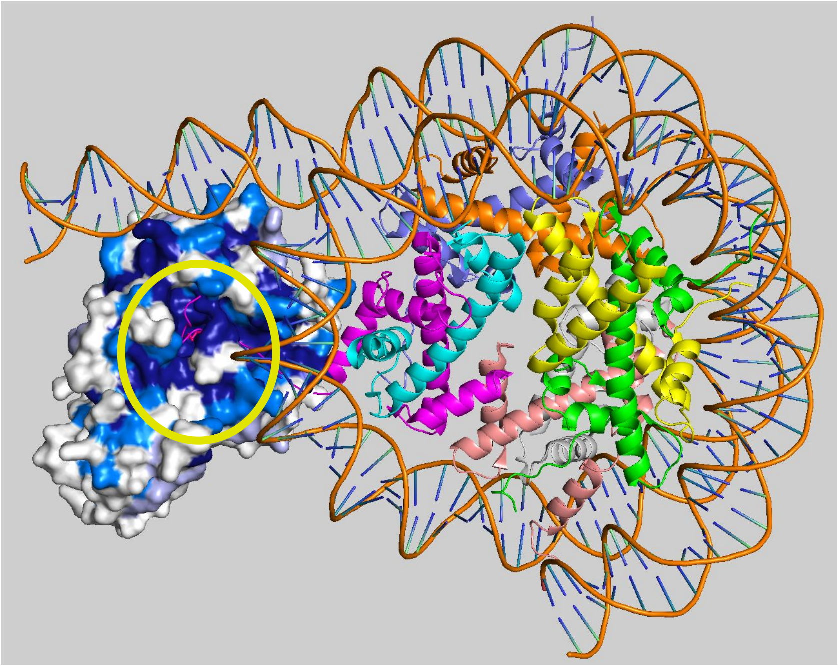

FBXL11 – PDB 7UV9

Magenta, Pink: Histone H3.2
Cyan, White: Histone H4
Green, Lavender: Histone H2A type 1
Yellow, Orange: Histone H2B type 1-C/E/F/G/I
Hot Pink molecule:
N-heptanoyl-N-hydroxy-beta-alanine ($C_{10}H_{19}NO_4$)
Orange ion: Fe^{3+}

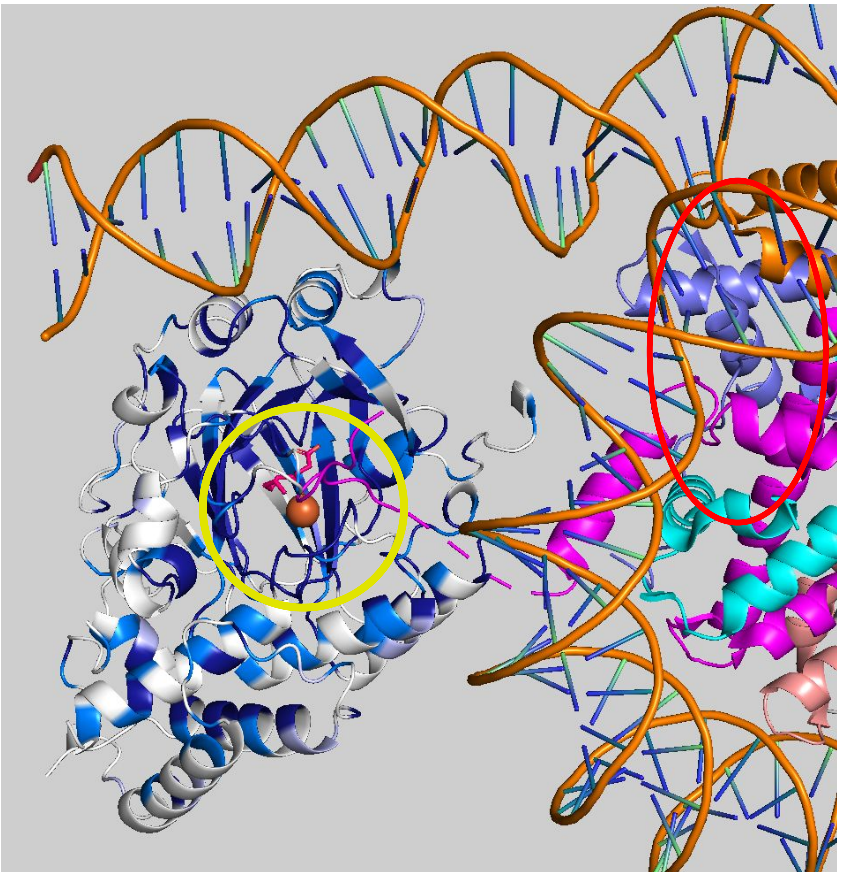

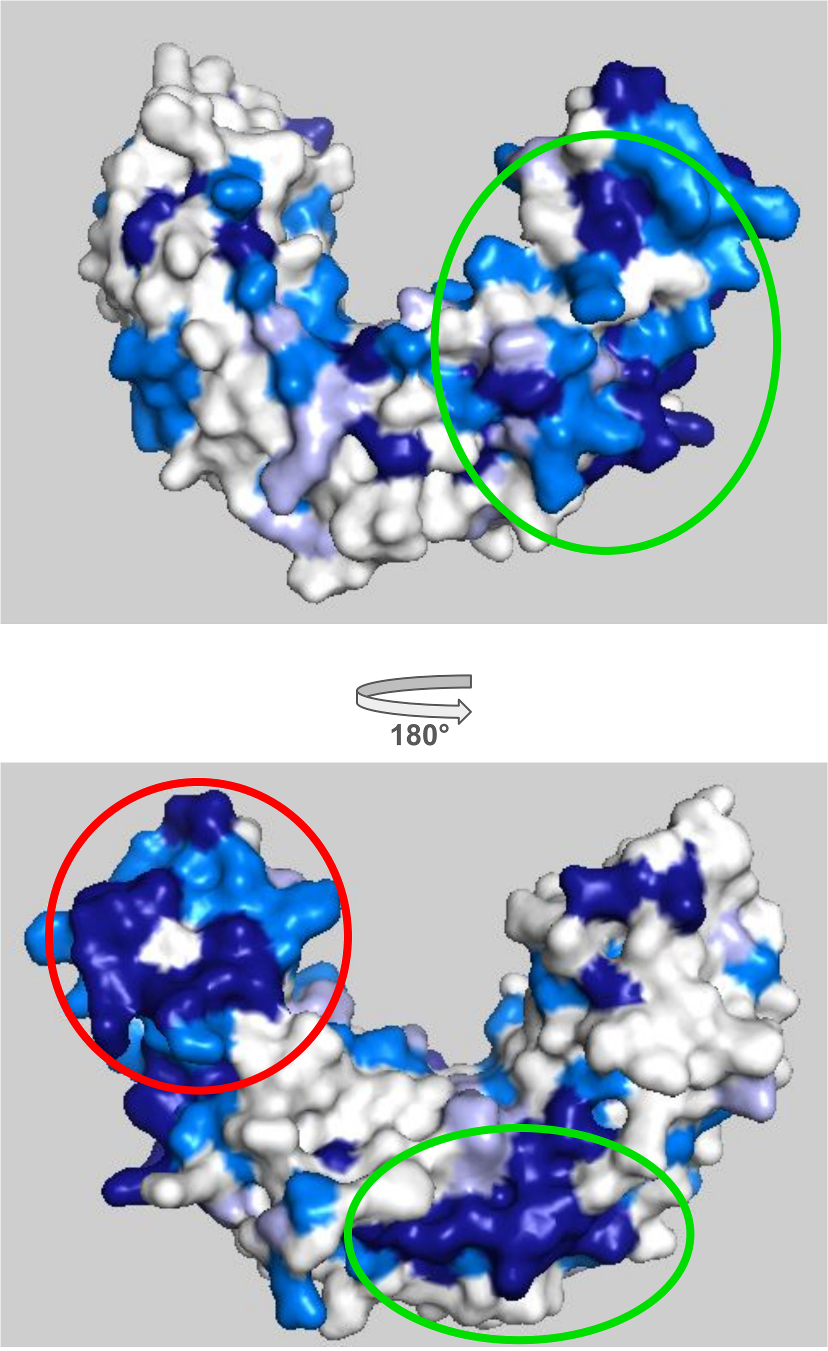

FBXL12

Run settings:

- % identity threshold: 40%
- % length variability threshold: 20%

Large, well conserved patch located on exterior face of the Fbox domain

Well conserved surfaces located on outer faces of LRR domain

FBXL13

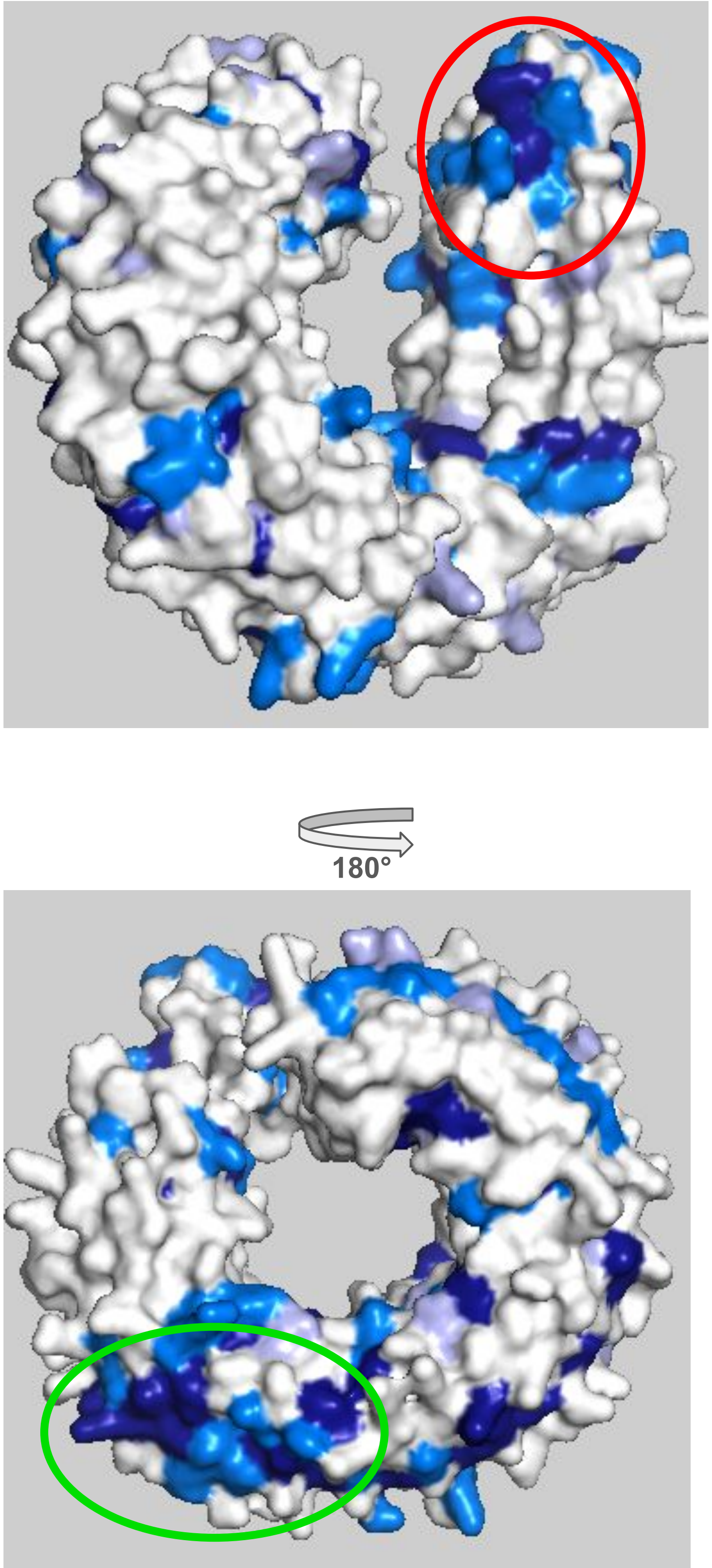

Run settings:

- % identity threshold: 40%
- % length variability threshold: 20%

Well conserved patch located on exterior face of the Fbox domain

Large, well conserved patch on external face of LRR domain

FBXL14

Run settings:

- % identity threshold: 40%
- % length variability threshold: 50%
- Removed species: Columba livia

Well conserved patch covering the Fbox domain

Large, well conserved patch on external face of LRR domain

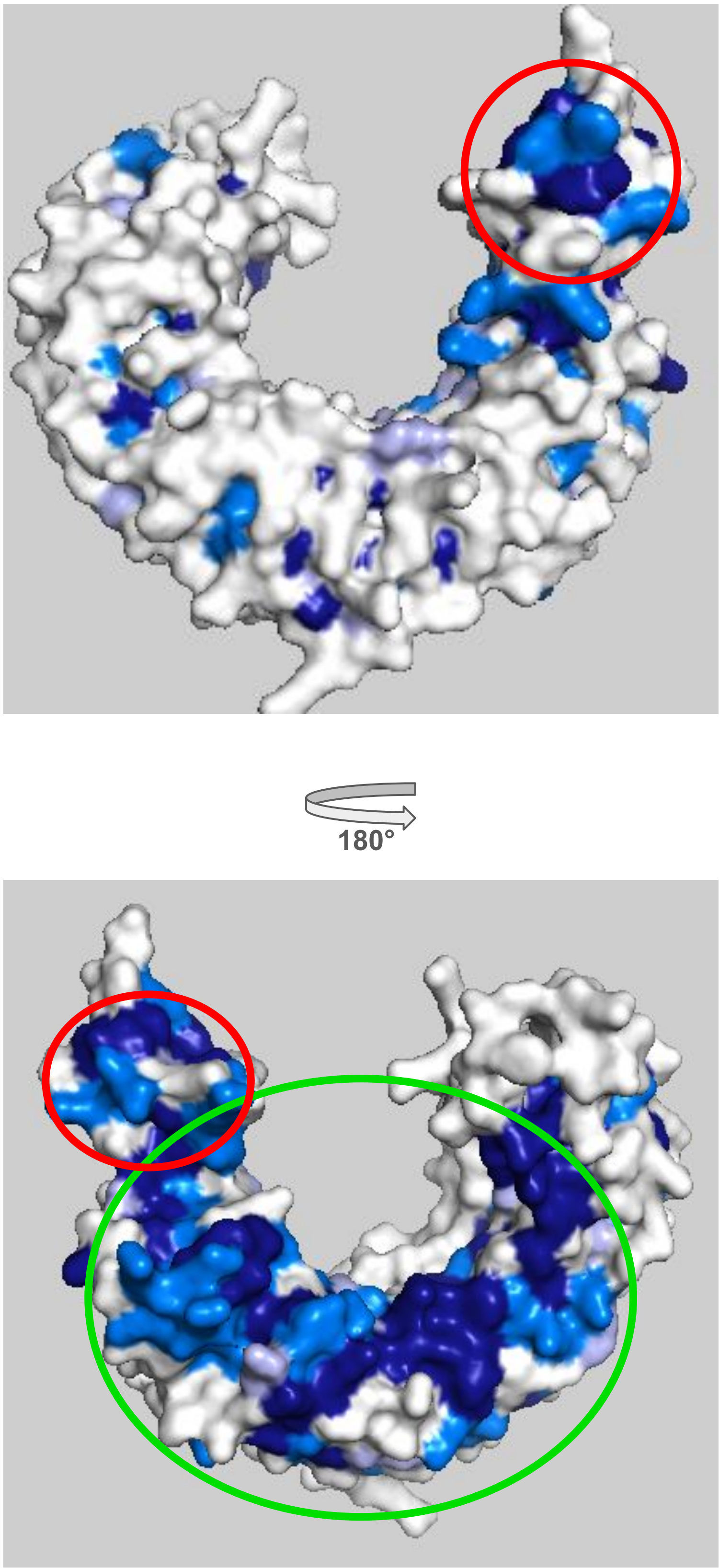

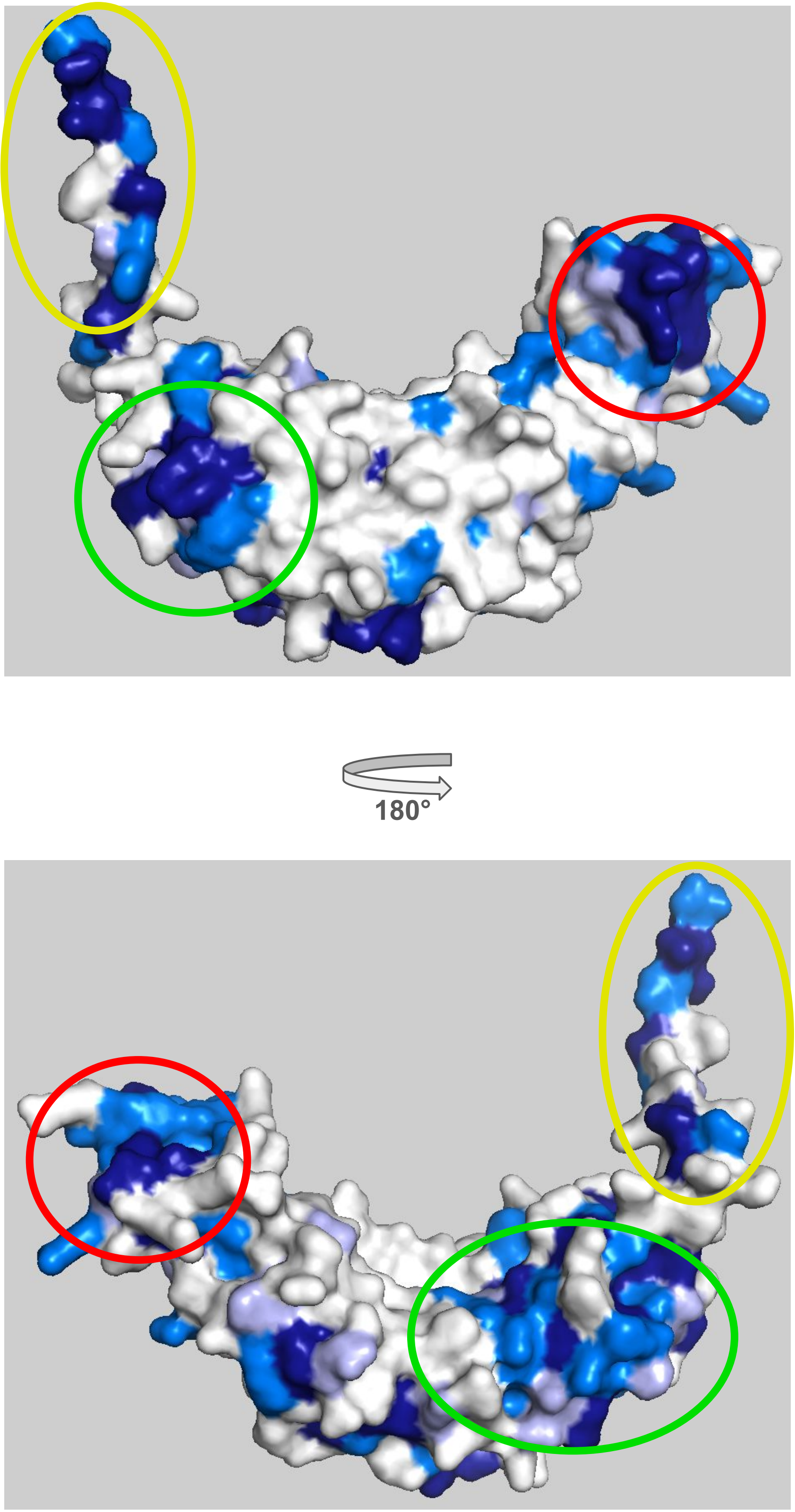

FBXL15

Run settings:
- % identity threshold: 40%
- % length variability threshold: 20%

Well conserved patch located on exterior face of the Fbox domain

Large, well conserved patch on external face of LRR domain

Short, well-conserved unstructured segment at the C-terminus

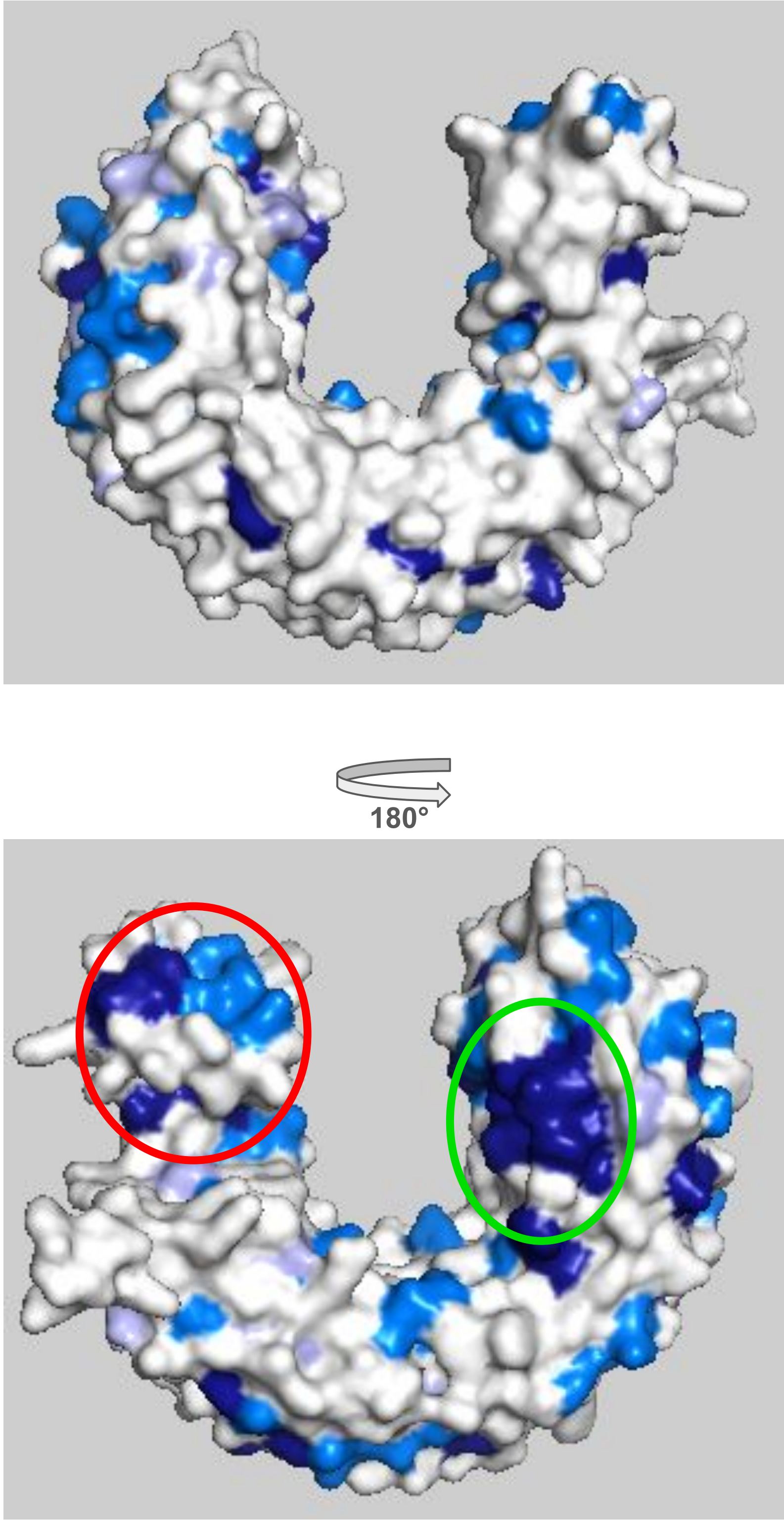

FBXL16

Run settings:
- % identity threshold: 40%
- % length variability threshold: 20%

Well conserved patch located on exterior face of the Fbox domain

Large, well conserved patch on internal face of LRR domain

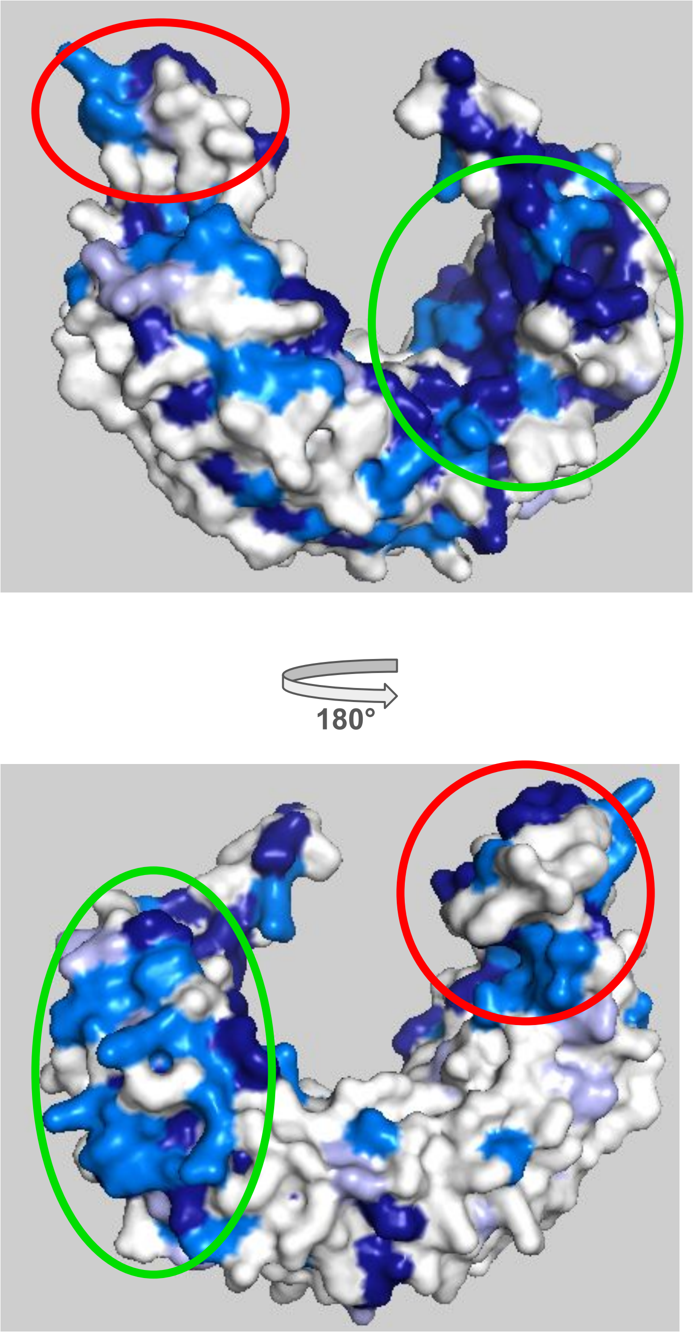

FBXL17

Run settings:

- % identity threshold: 40%
- % length variability threshold: 50%

Well conserved patch located on exterior face of the Fbox domain

Large, well conserved surface on the end of the LRR domain

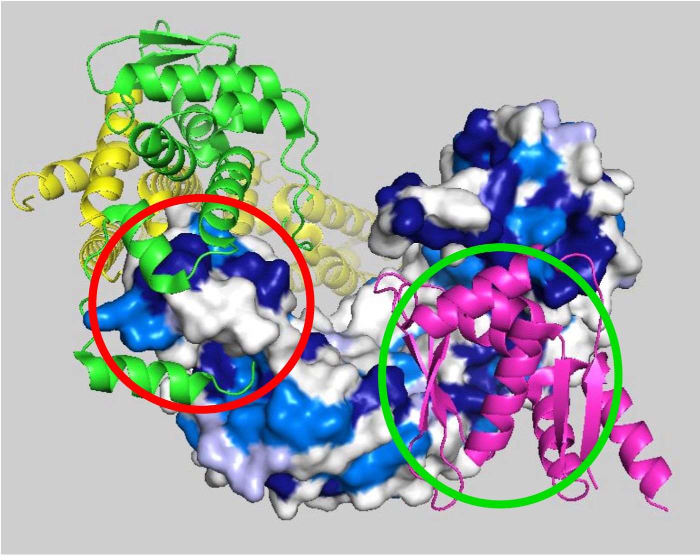

FBXL17 – PDB 6WCQ

Green: SKP1
Magenta: KEAP1 (Kelch-like ECH-associated protein 1)
Yellow: CUL1 (Cullin-1)

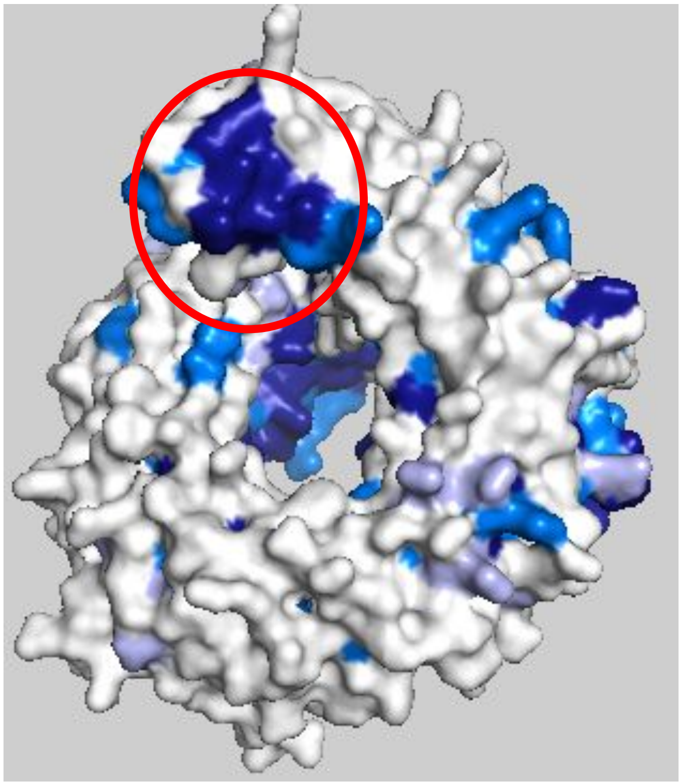

FBXL18

Run settings:

- % identity threshold: 40%
- % length variability threshold: 20%

Well conserved patch located on exterior face of the Fbox domain

Large, well conserved patch on one side of LRR domain

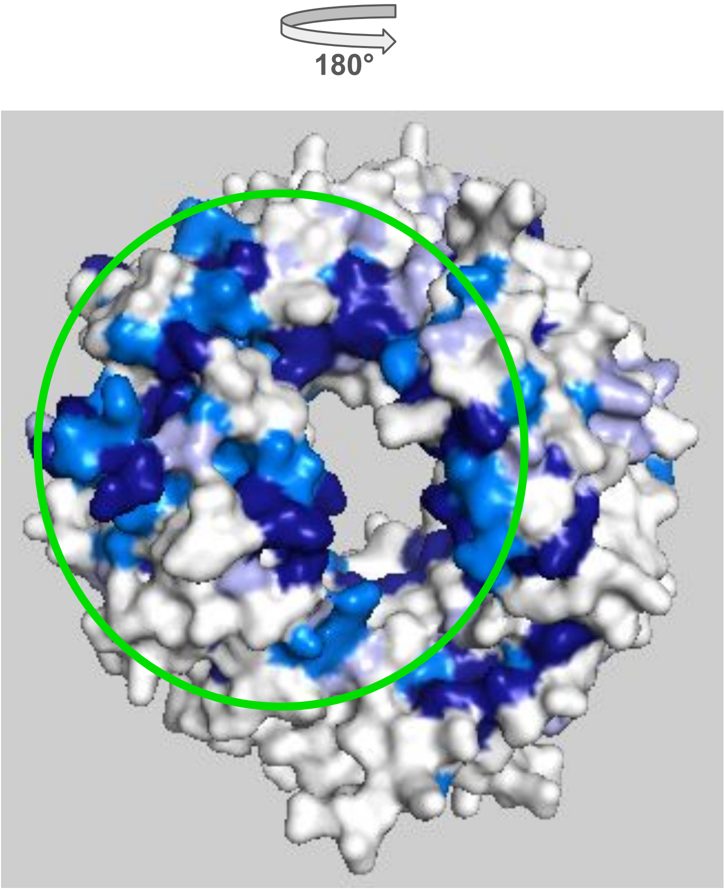

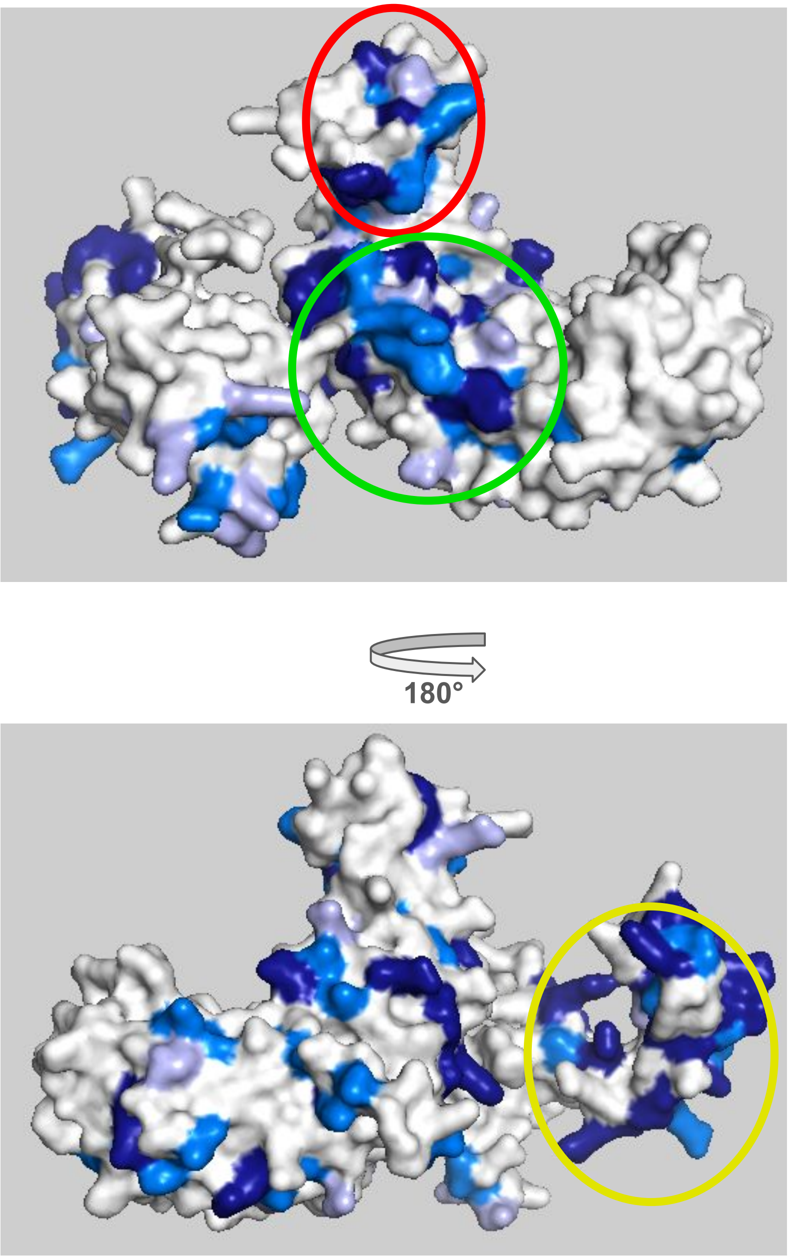

FBXL19

Run settings:
- % identity threshold: 30%
- % length variability threshold: 30%

Small, well conserved patch located on exterior face of the Fbox domain

Large, well conserved patch located on side of the LRR domain

Well conserved patch located in cleft of the CXXC-type zinc finger domain

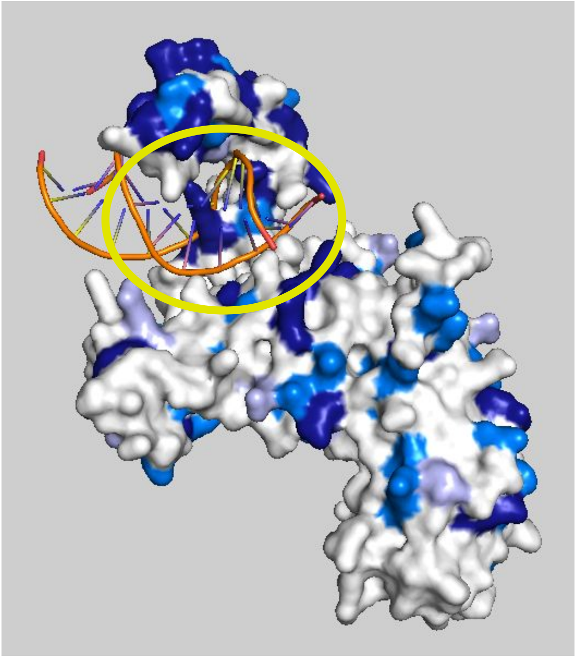

FBXL19 – PDB 6ASB

Orange: DNA

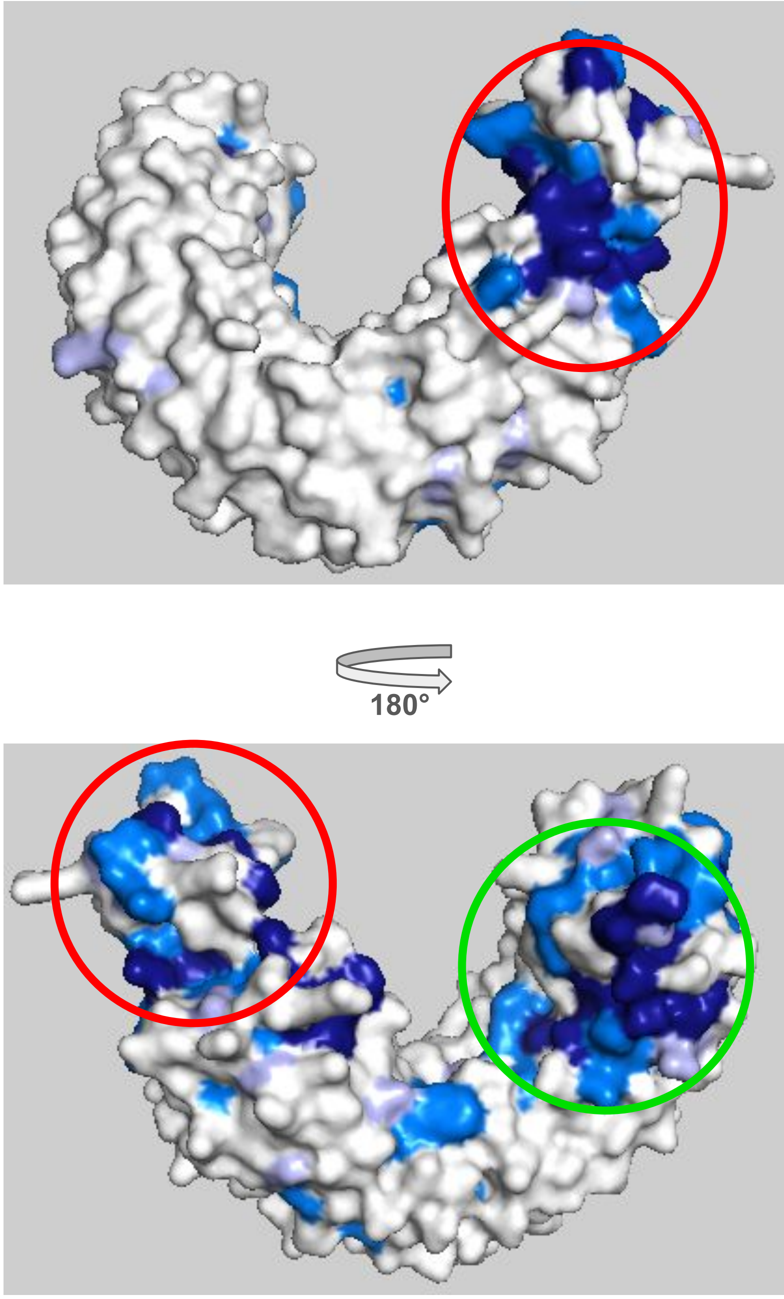

FBXL20

Run settings:

- % identity threshold: 40%
- % length variability threshold: 20%

Well conserved patch located on exterior face of the Fbox domain

Large, well conserved patch located on inner face of the LRR domain

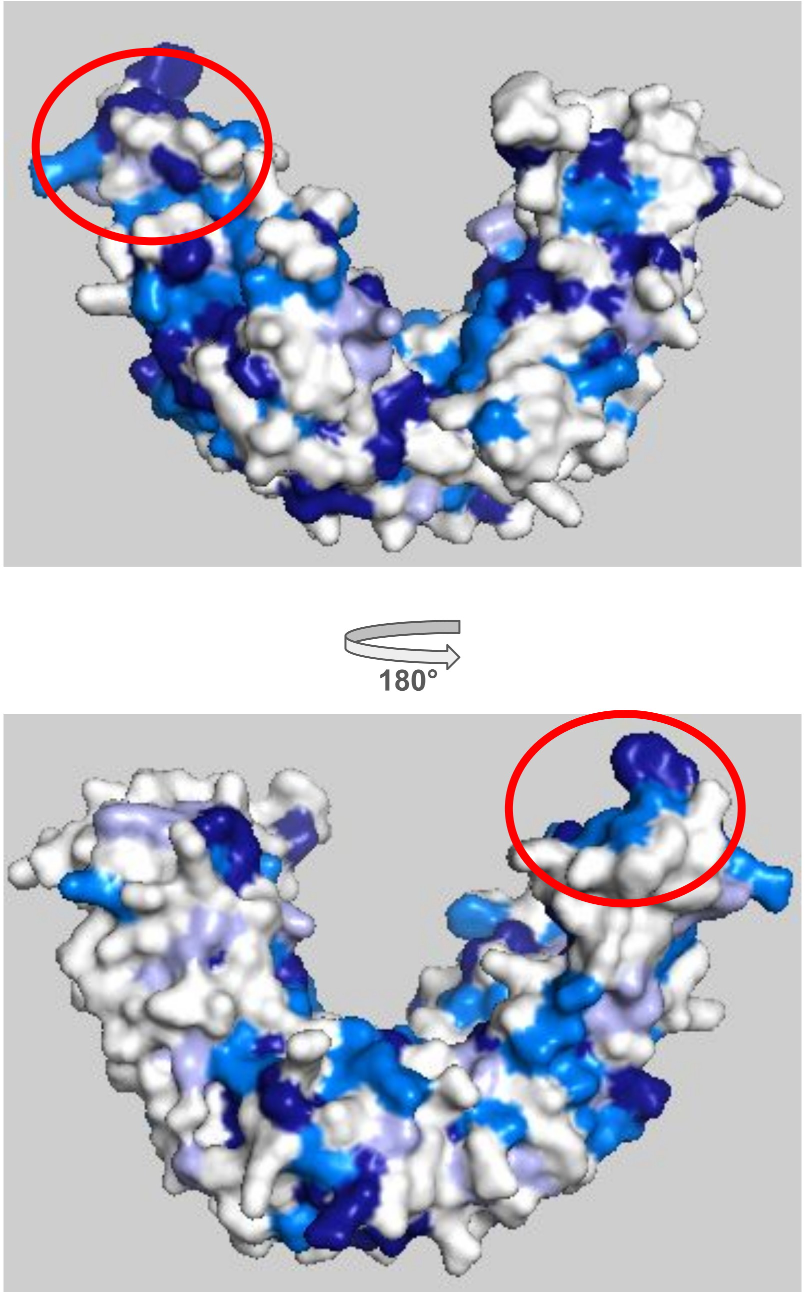

FBXL21P

Run settings:

- % identity threshold: 40%
- % length variability threshold: 20%

Conserved patch on Fbox domain

No significant hotspots located. Mottled conservation present across whole protein surface

Note: According to UniProt, this protein could be the product of a pseudogene, which may explain spotty results.

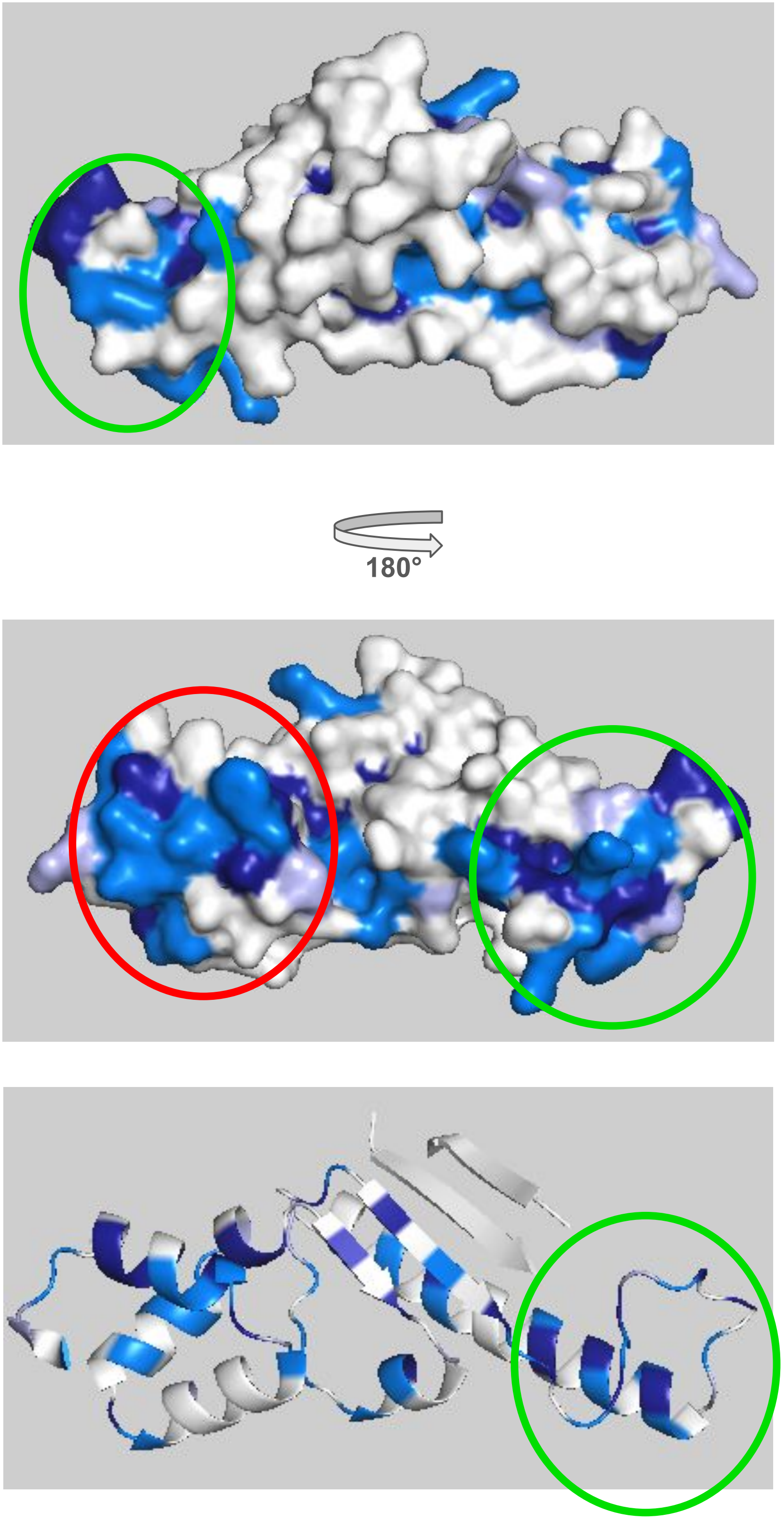

FBXL22

Run settings:

- % identity threshold: 40%
- % length variability threshold: 65%

Well conserved patch located on exterior face of the Fbox domain

Large, well conserved patch located on unstructured loop and alpha helix

FBXW Hotspots

FBXW1A (BTRC)

Run settings:

- % identity threshold: 40%
- % length variability threshold: 20%

Well conserved surface covering Fbox domain

Large, perfectly conserved surface covering front face and interior of WD repeat domain

Well-conserved interior faces of D-domain (dimerization)

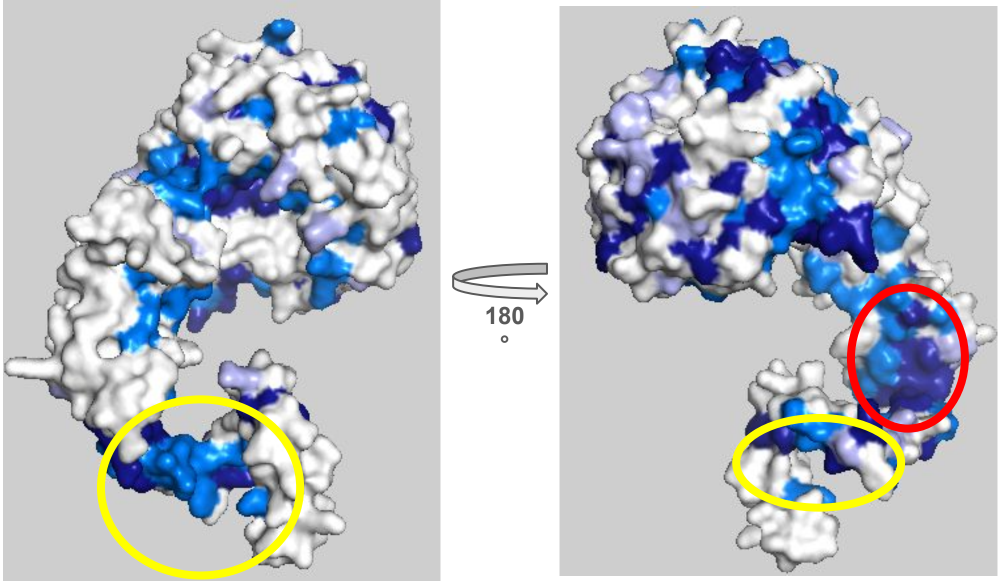

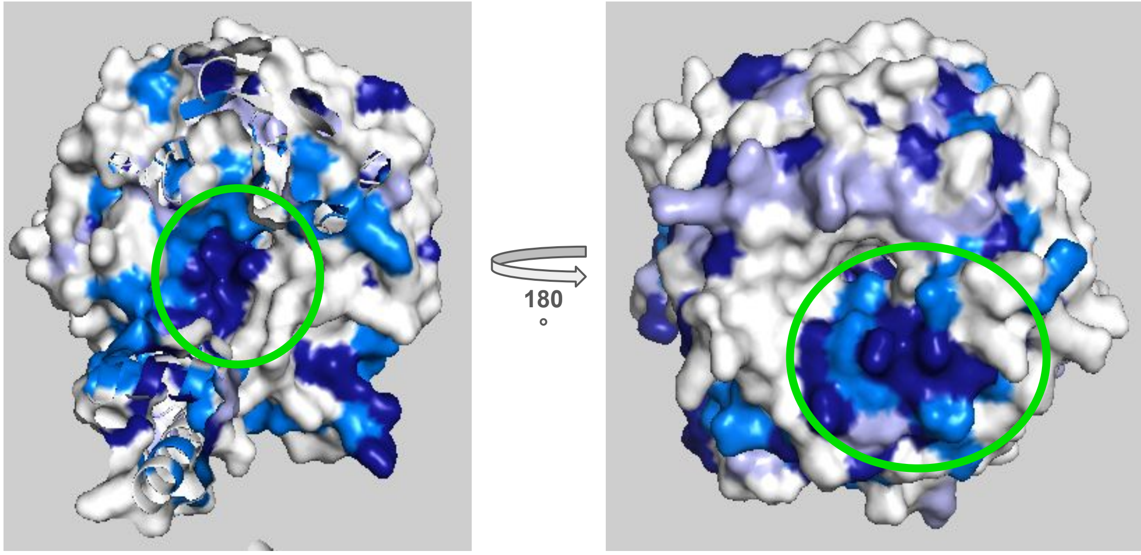

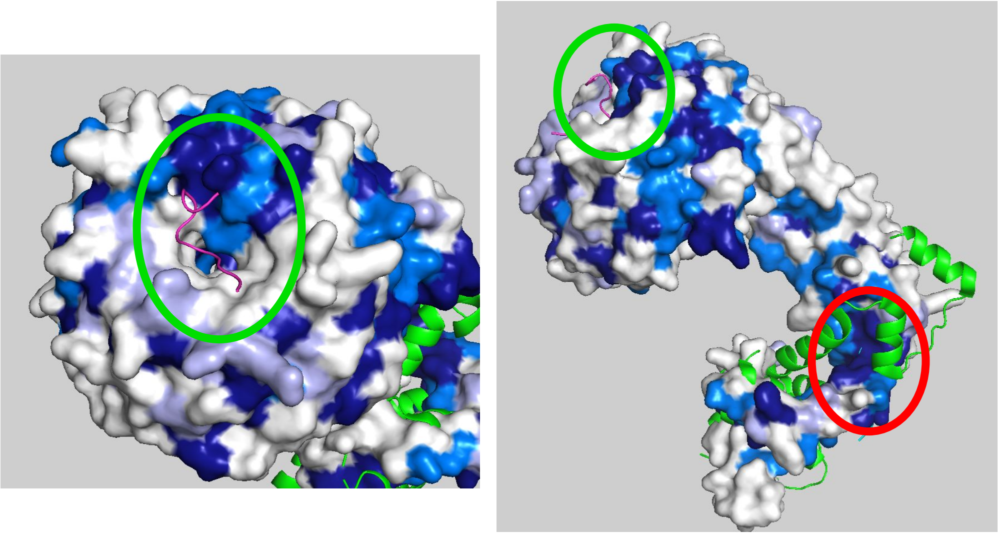

FBXW1A (BTRC) – PDB 6M91

Green: SKP1
Magenta: Catenin beta-1

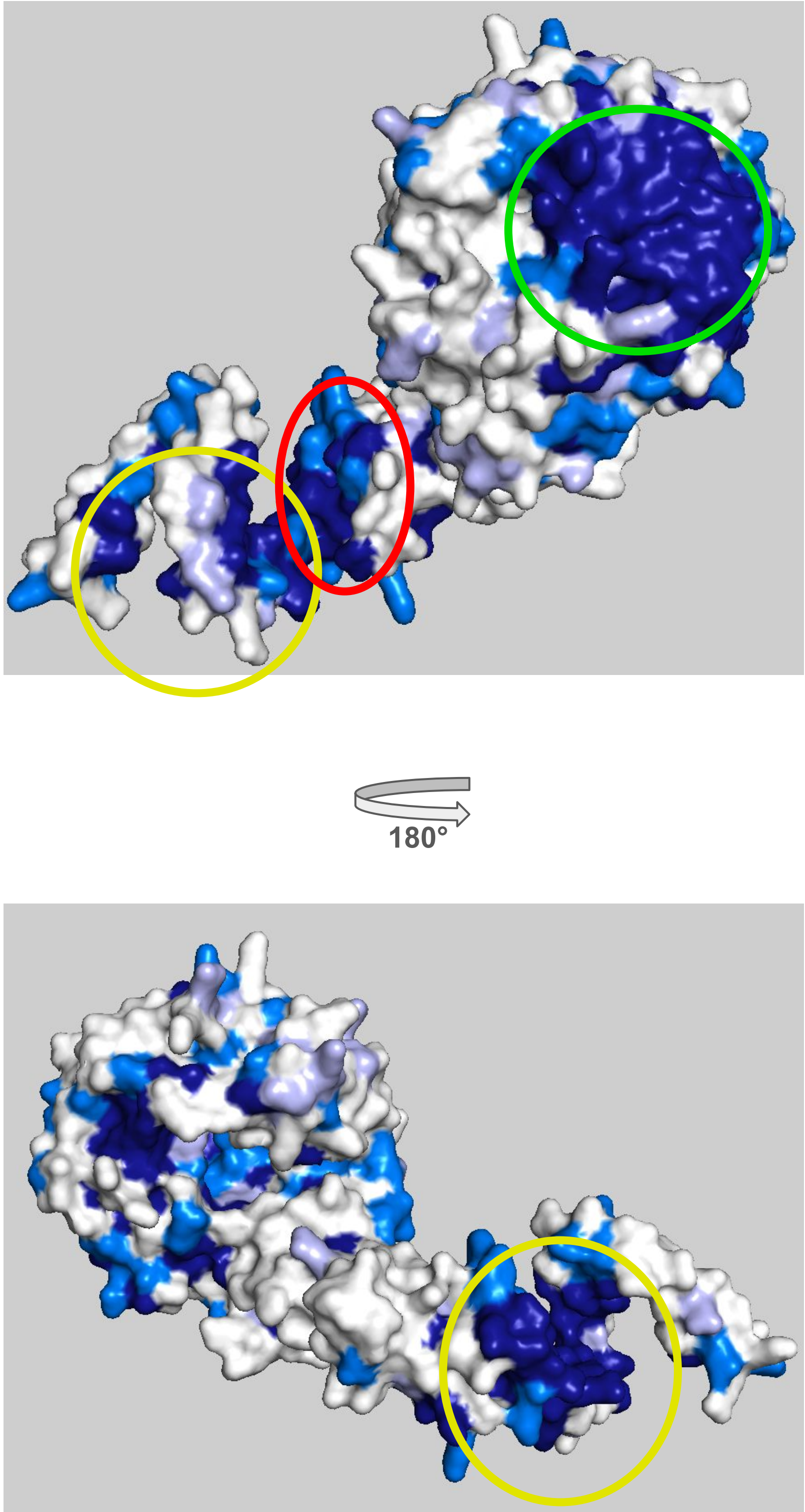

FBXW2

Run settings:

- % identity threshold: 50%
- % length variability threshold: 20%
- Removed species: Lytechinus variegatus, Eptatretus burgeri

Perfectly conserved region covering the Fbox domain

Large, perfectly conserved patch covering front face of WD repeat domain

Large, perfectly conserved region covering inner faces of 3 alpha helices forming N-terminal coil-shaped domain. AlphaFold predictions show this is likely a dimerization site

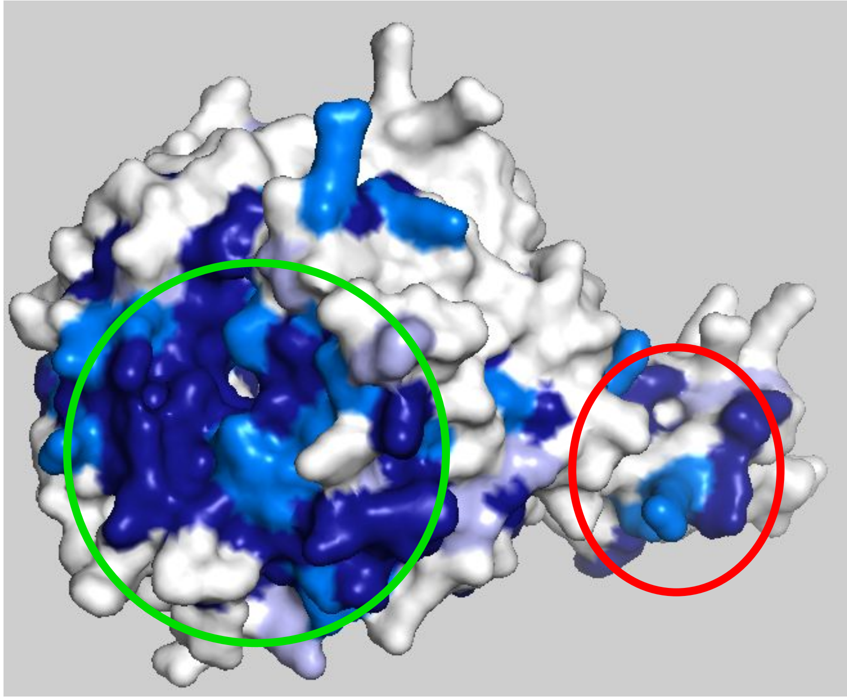

FBXW4

Run settings:

- % identity threshold: 50%
- % length variability threshold: 20%

Well conserved region located on the Fbox domain

Large, perfectly conserved patch covering front face of WD repeat domain

180°

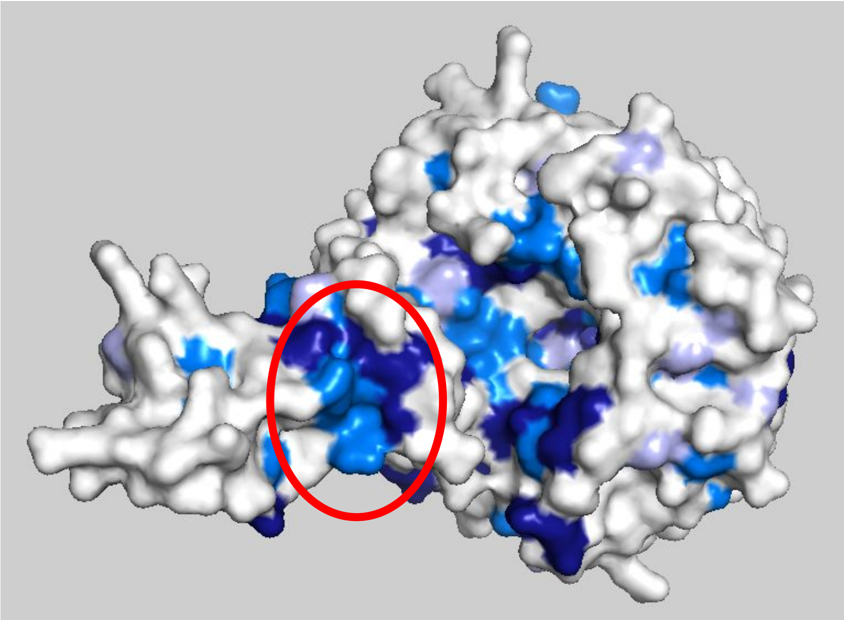

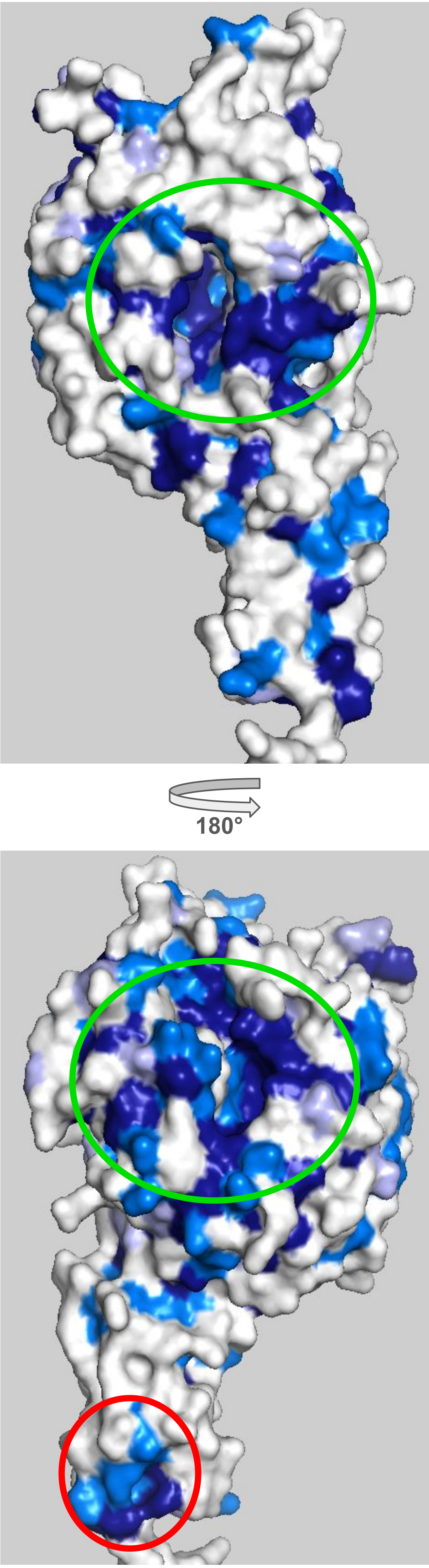

FBXW5

Run settings:

- % identity threshold: 50%
- % length variability threshold: 20%
- Removed species: Pogona vitticeps, Columba livia

Well conserved region covering the Fbox domain

Large, perfectly conserved patch covering front face of WD repeat domain

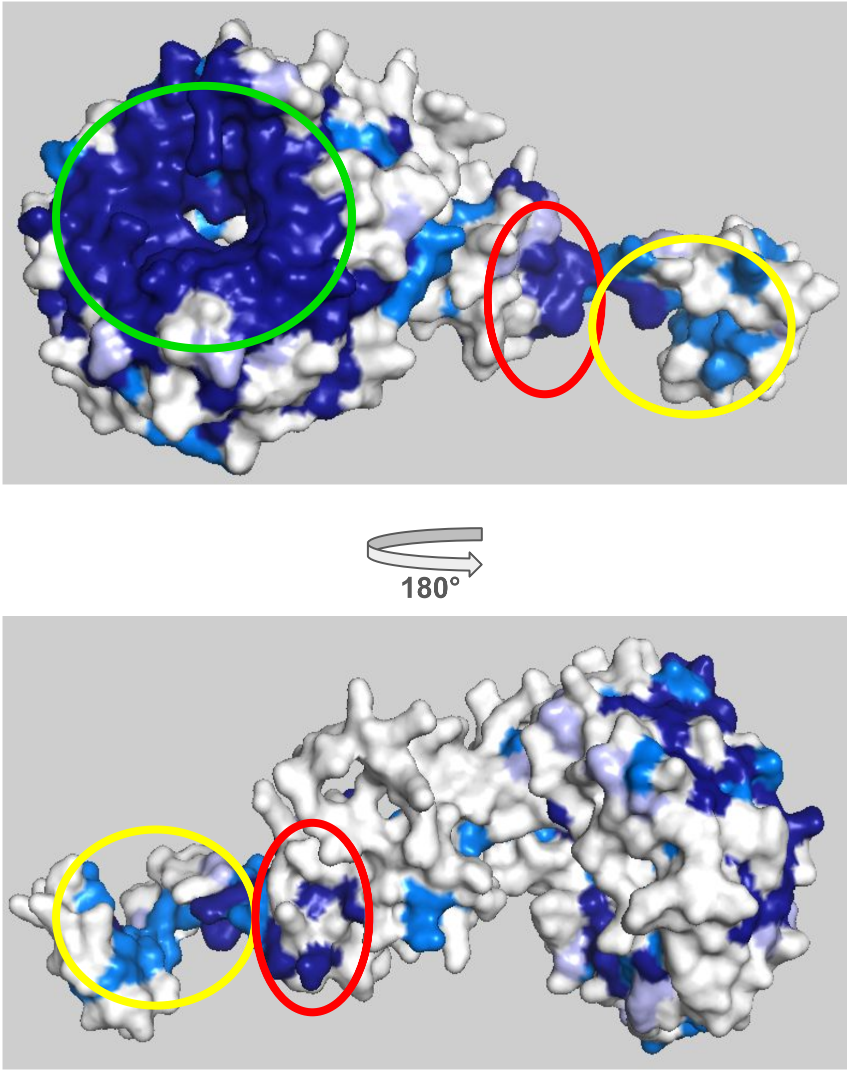

FBXW7

Run settings:

- % identity threshold: 50%
- % length variability threshold: 50%

Well conserved surface covering Fbox domain

Large, perfectly conserved surface covering front face of WD repeat domain

Well-conserved interior faces of D-domain (dimerization)

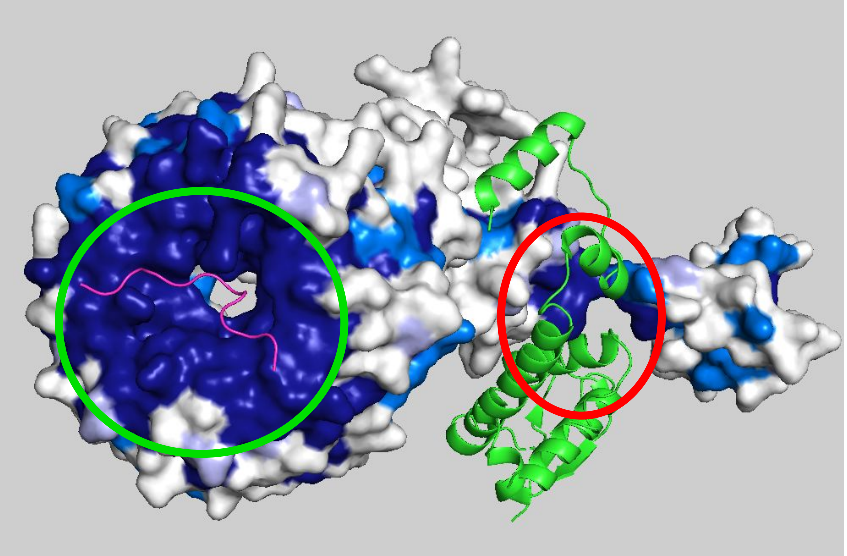

FBXW7 – PDB 7T1Z

Green: SKP1
Magenta: c-Myc N terminal degron

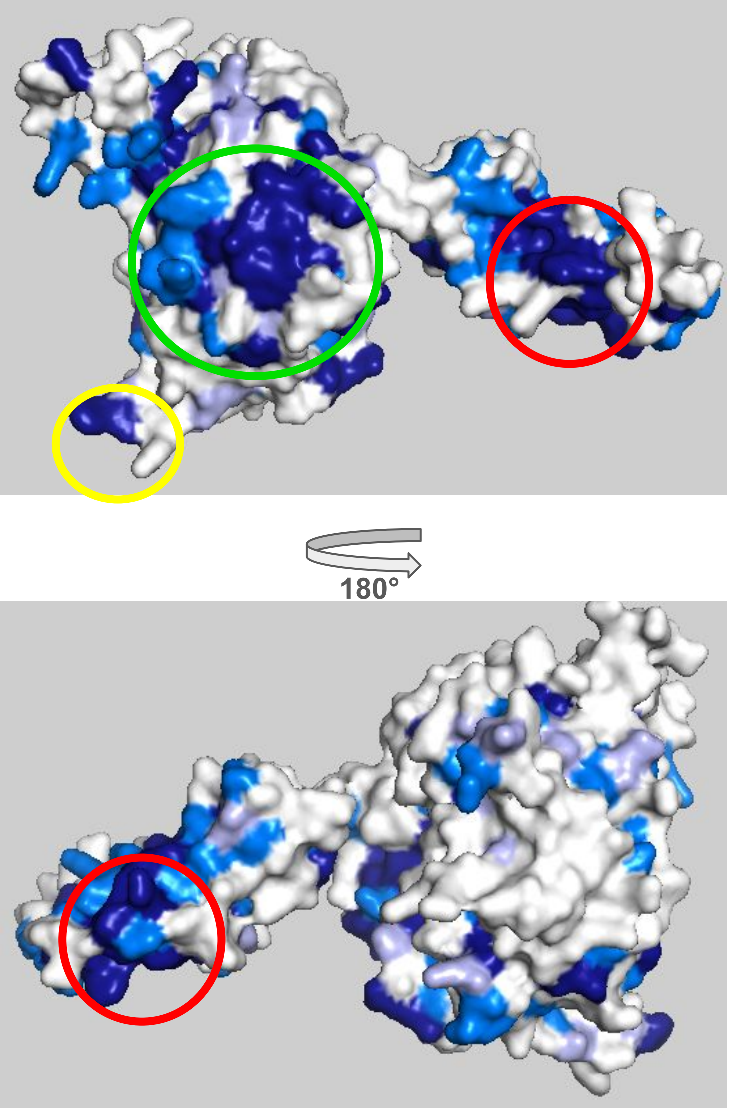

FBXW8

Run settings:

- % identity threshold: 45%
- % length variability threshold: 20%
- Removed species: Pogona vitticeps, Columba livia

Well conserved surface covering Fbox domain

Large, perfectly conserved surface covering side face of WD repeat domain

Single perfectly-conserved residue on unstructured loop

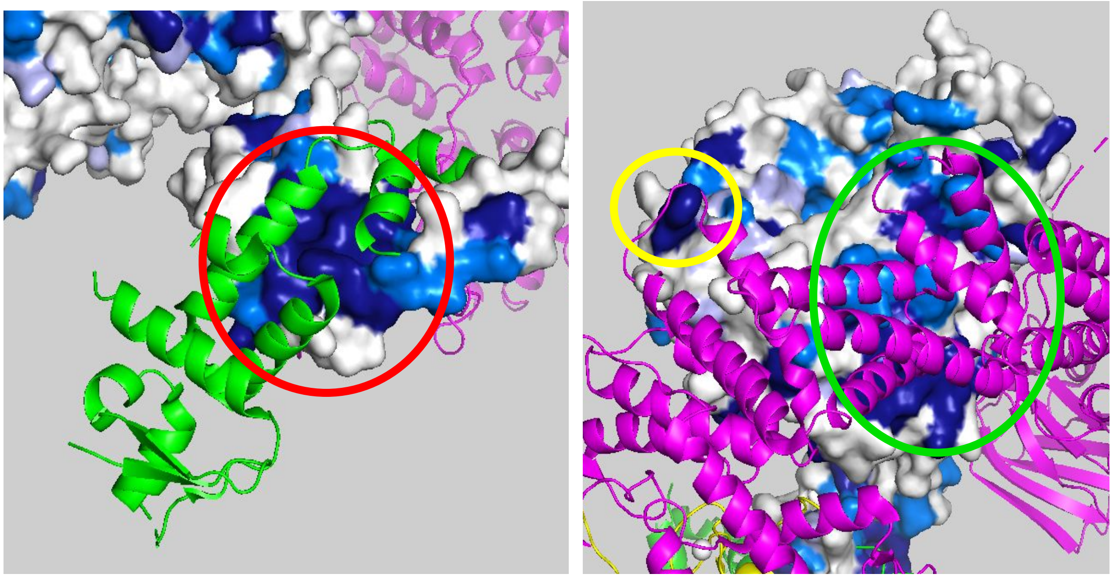

FBXW8 – PDB 7Z8B

Green: SKP1
Magenta: CUL7

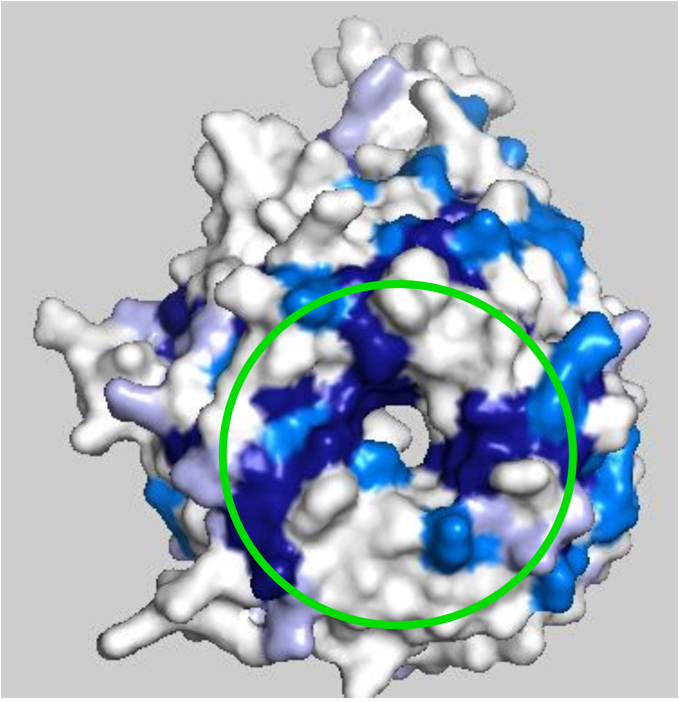

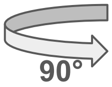

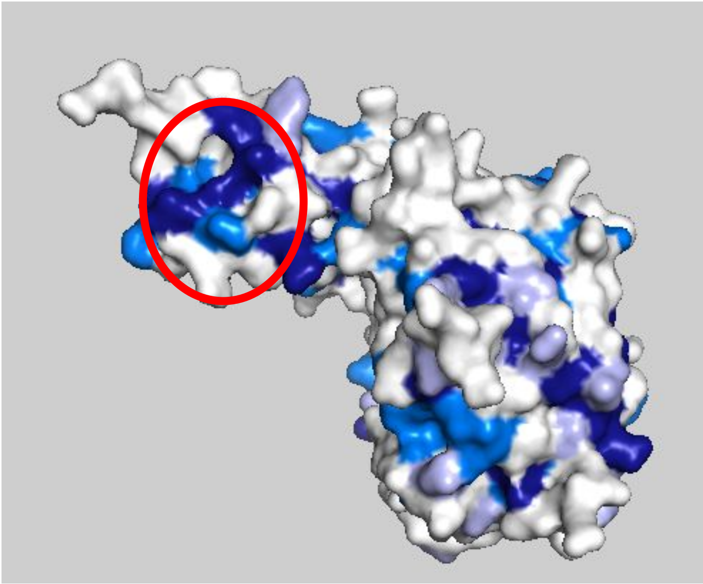

FBXW9

Run settings:

- % identity threshold: 50%
- % length variability threshold: 50%

Well conserved surface covering Fbox domain

Large, well conserved surface covering front face of WD repeat domain

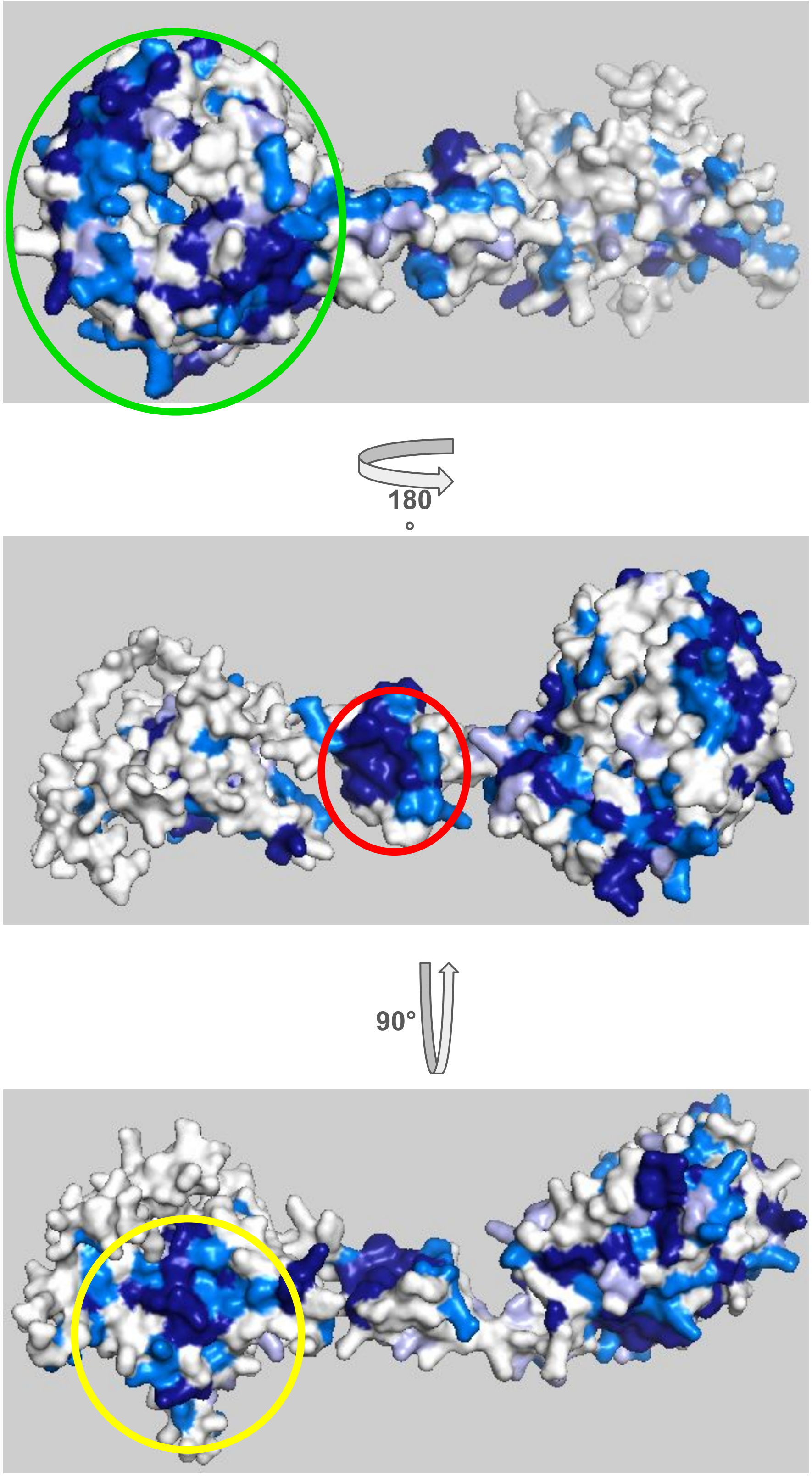

FBXW10

Run settings:

- % identity threshold: 40%
- % length variability threshold: 50%

Well conserved surface covering Fbox domain

Large, perfectly conserved surface covering front face and sides of WD repeat domain

Large, well conserved patch on one face of unnamed N-terminal domain

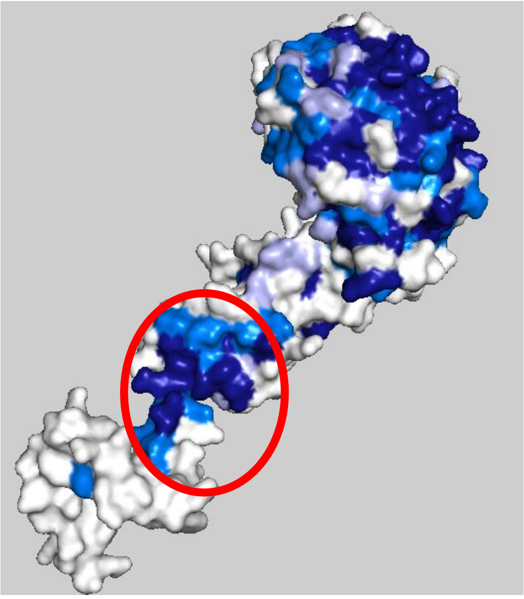

FBXW11

Run settings:

- % identity threshold: 50%
- % length variability threshold: 20%

Well conserved surface covering Fbox domain

Large, perfectly conserved surface covering front face and interior of WD repeat domain

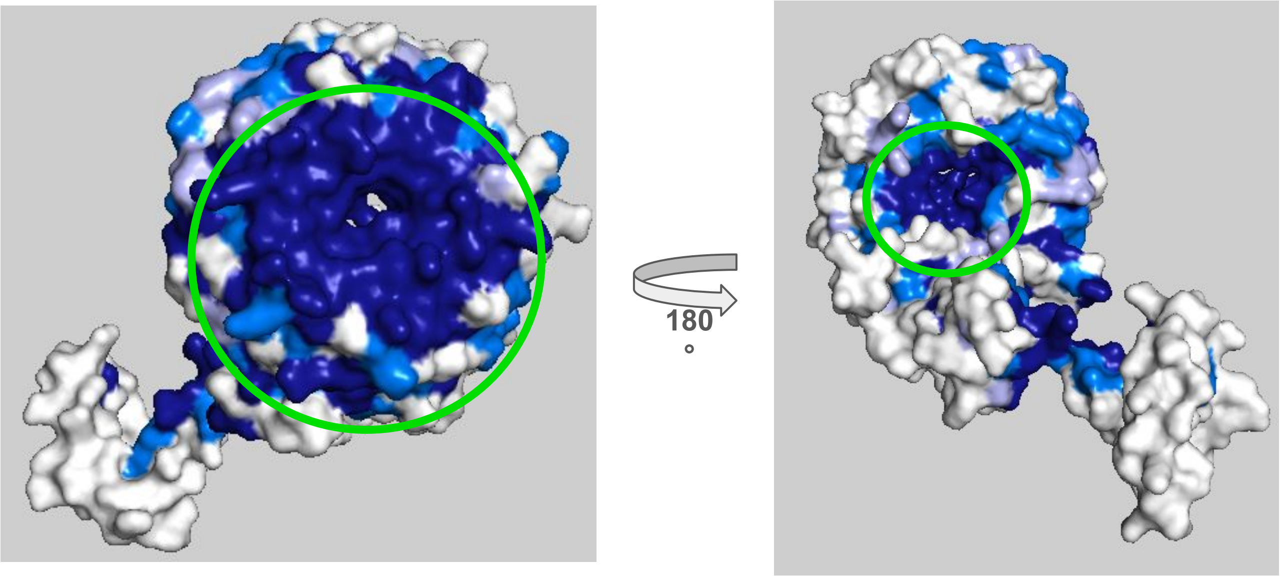

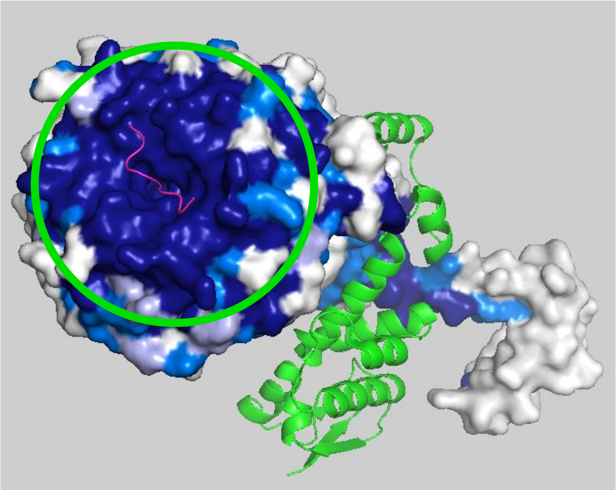

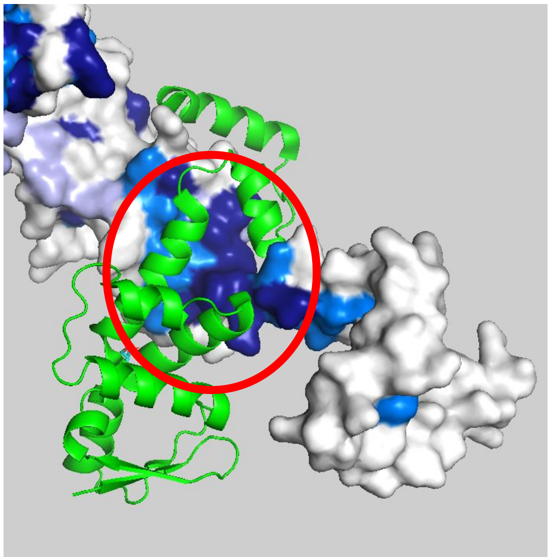

FBXW11 – PDB 6WNX

Green: SKP1
Magenta: Catenin beta-1

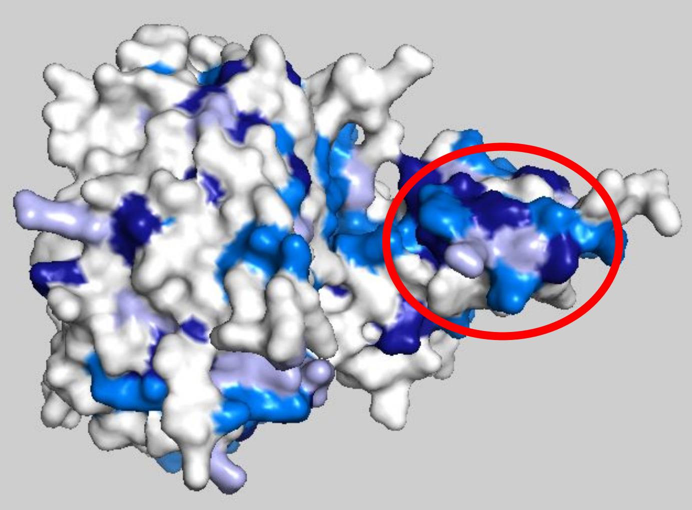

FBXW12

Run settings:

- % identity threshold: 40%
- % length variability threshold: 50%

Conserved surface covering Fbox domain

No other significant hotspots located. Mottled conservation present across much of protein surface

90°

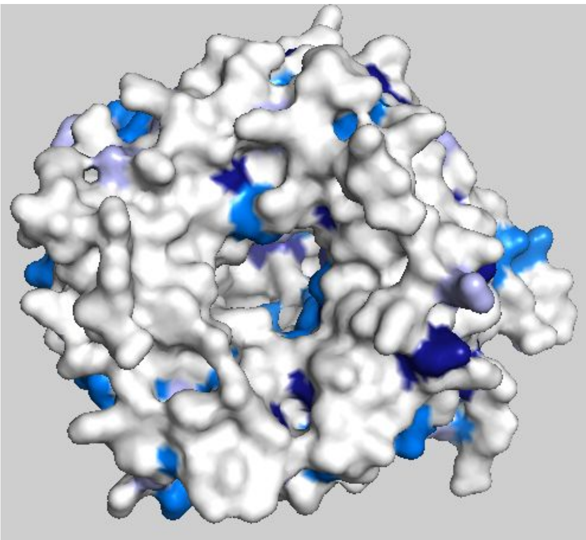

Interesting note: This protein seems to have abnormally low similarity with most species, even among mammals.
On 40% identity threshold and 50% length threshold, only 9 non-human species make it into the alignment, and only 1 of them has % similarity higher than 80.

```
Ursus_arctos_[51]                 SLC-NFS-GCL-SQTWKRLFLQQTKLEHRMAAVKPEDFLFR--------- 87
Canis_lupus_[58]                  SFC-NFS-QCLGSLTWKQFFLKQRKQEYRMALAKPEDFTFREASGNLGIL 98
Felis_catus_[60]                  SFC-NFS-KCLGSQTWKQFFLKQTKQEYQMALAQPEDFIFKEATGNLGII 98
Neosciurus_carolinensis_[58]      RFC-NFTYERLGTQTWKQLFLHQTKQERRMACAQSEDFIYKEAAENLGME 99
Myotis_myotis_[49]                SIS-WEG---LQVPSWKQLFLTQARRERCMMGAQPQDFAYREATGG---- 92
Homo_sapiens_[100]                DCS-NFTNQHLGTHTWKQFFLHQRRKELRLALAQPHNFIYK-VTKNIAFE 98
Macaca_mulatta_[86]               DCS-NFTNQHLGTHTWKKFFLHQRRKELRLALAQPHNFIYK-VTKNIAYD 98
Danio_rerio_[41]                  LYCYMFTLPLHGLETWKQFIFDRTWQEHAKTRAKPEDFTYKEFPVEFEFR 100
Mus_musculus_[42]                 LFCDMVTLQLLGTETWKQFFVFRTWQEHAKTRAKPEDFTYKEIPAEYGFR 100
Rattus_norvegicus_[42]            FFYEVVTLELLGTESWKQFFVCRTRQERAKSRAKPEDFTYKEIPVEIGAP 100
STRUCTURE [7Z8B]:                 XXX XXXXXXXXXXXXXXXXXXXXXXXXXXXXXXXXXXX XXXXXXXX
ALPHAFOLD STRUCTURE:              --- ---AAAA----AAAAAAAAAAAAAAAAA-----BBBB BBBBB---
                                                :**:::. :   *     .:..:* ::
```

90°

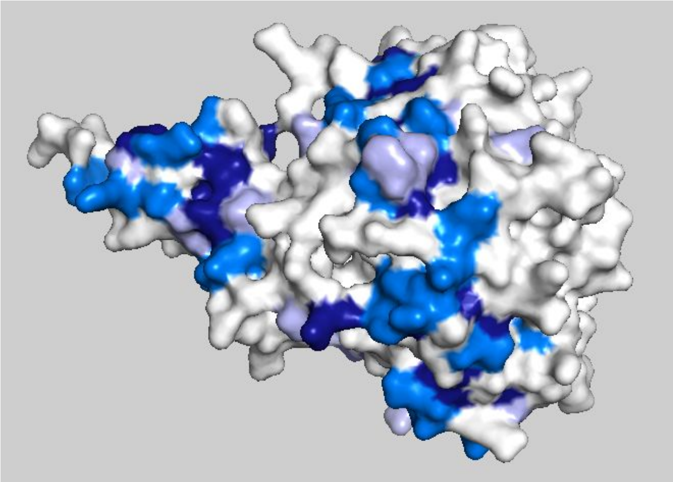

Supplement: Supplementary file 3 — Supplementary material [file mmc3.pdf]
